# Molecular Graphene Nanoribbon Junctions

**DOI:** 10.1021/jacs.3c11340

**Published:** 2024-02-02

**Authors:** Mauro Marongiu, Tracy Ha, Sara Gil-Guerrero, Kavita Garg, Marcos Mandado, Manuel Melle-Franco, Ismael Diez-Perez, Aurelio Mateo-Alonso

**Affiliations:** † POLYMAT, University of the Basque Country UPV/EHU, Avenida de Tolosa 72, 20018 Donostia-San Sebastian, Spain; ‡ Department of Chemistry, Faculty of Natural & Mathematical Sciences, King’s College London, Britannia House, 7 Trinity Street, SE1 1DB London, United Kingdom; § CICECO—Aveiro Institute of Materials, Department of Chemistry, University of Aveiro, 3810-193 Aveiro, Portugal; ∥ Department of Physical Chemistry, University of Vigo, Lagoas-Marcosende s/n, 36310 Vigo, Spain; ⊥ Ikerbasque, Basque Foundation for Science, 48009 Bilbao, Spain

## Abstract

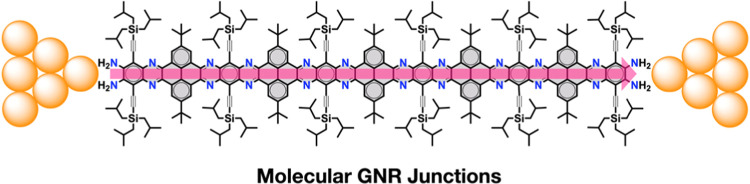

One of the challenges for the realization of molecular electronics is the design of nanoscale molecular wires displaying long-range charge transport. Graphene nanoribbons are an attractive platform for the development of molecular wires with long-range conductance owing to their unique electrical properties. Despite their potential, the charge transport properties of single nanoribbons remain underexplored. Herein, we report a synthetic approach to prepare N-doped pyrene-pyrazinoquinoxaline molecular graphene nanoribbons terminated with diamino anchoring groups at each end. These terminal groups allow for the formation of stable molecular graphene nanoribbon junctions between two metal electrodes that were investigated by scanning tunneling microscope-based break-junction measurements. The experimental and computational results provide evidence of long-range tunneling charge transport in these systems characterized by a shallow conductance length dependence and electron tunneling through >6 nm molecular backbone.

## Introduction

The development of molecular electronics requires materials that enable long-range charge transport. Single-molecule charge transport studies have shown good promise for π-conjugated molecules as molecular wires displaying long-range conductance properties.^1−6^ These include oligoenes,^7,8^ oligomethines,^9,10^ oligoynes,^11,12^ cumulenes,^13,14^*p*-phenylene radicals,^15^ acenes,^16−19^ and porphyrin tapes,^20,21^ among others.^22,23^ Several studies have shown that coherent tunneling dominates over short distances, whereas incoherent hopping dominates over long distances. This tunneling-to-hopping transition has been reported so far to vary approximately between 2.7 and 5.6 nm depending on the electronic structure of the molecular wire.^24−33^ Most importantly, some of these studies have correlated larger tunneling-to-hopping transition values for planarized conjugated backbones.^30,31,33^ This opens up a large number of possibilities for systems made out of fused aromatic rings that extend in one-dimension (1D), such as graphene nanoribbons (GNRs).^34−45^ In fact, GNRs are an attractive platform for studying long-range electronic transport, given their broad structural and electronic diversity. For instance, the electronic properties of GNRs are determined by their edge structure, width, length, and heteroatom-doping. However, despite their potential and the impressive recent advances in the synthesis of monodisperse (or molecular)^36,37,39,46−68^ and polydisperse GNRs,^34−36,38,40−44,69^ the charge transport properties of single GNRs remain underexplored^70−74^ and, in turn, the theoretical transport models for GNRs remain unverified. Single GNR charge transport studies have been carried out almost exclusively on in situ-synthesized GNRs,^70−74^ and only a few experimental studies have been reported on ex situ-synthesized GNRs if work on 2 nm long heteroacenes is considered.^16−19^ Therefore, expanding our abilities to investigate *ex situ*-synthesized GNRs will pave the way to directly investigating GNRs synthesized in solution and allow correlating results between complementary techniques.

**Figure 1:**
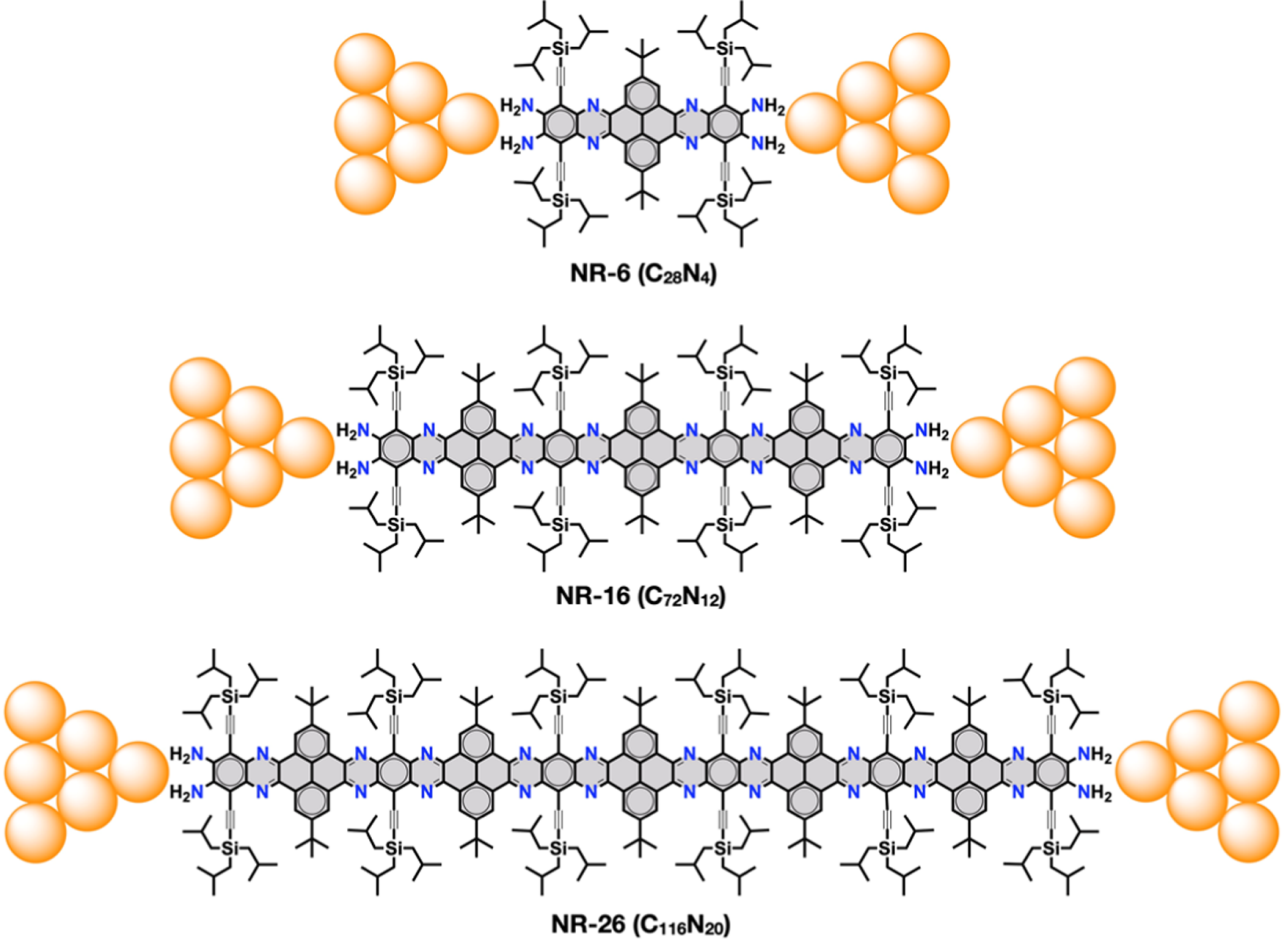
Molecular GNR junctions studied in this work (the molecular formula indicates only the atoms in the aromatic core, highlighted in gray).

**Scheme 1:**
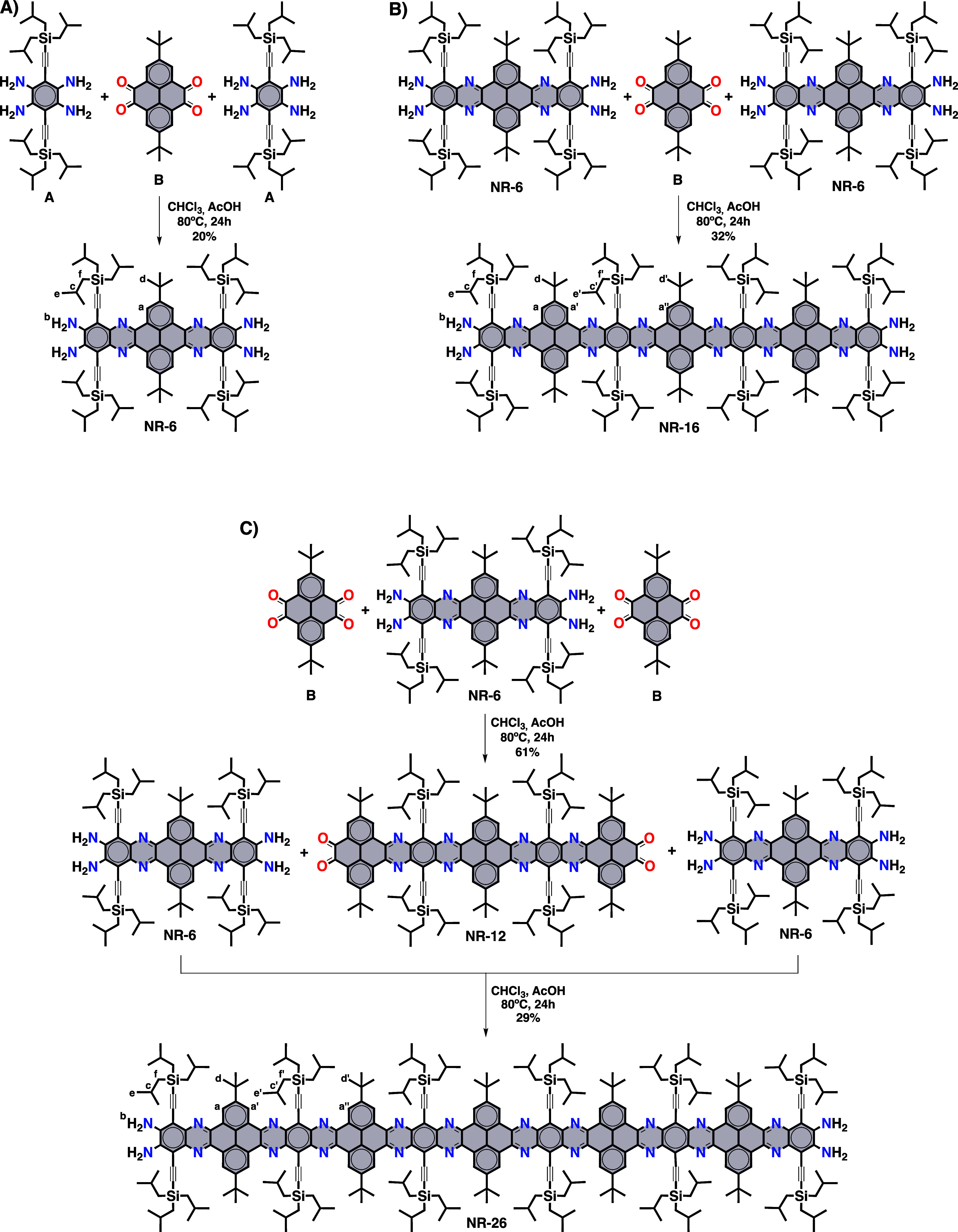
Synthesis of (A) **NR-6**, (B) **NR-16**, and (C) **NR-26**.

Herein, we report the ex situ synthesis and single-molecule charge transport studies of a new series of pyrene-pyrazinoquinoxaline molecular GNRs, namely, **NR-6**, **NR-16**, and **NR-26** (Figure 1). These nitrogen-doped molecular GNRs show, respectively, 1.7, 4.1, and 6.5 nm long backbones displaying high stability, thanks to their hybrid zigzag-cape edge structure. The GNRs have been synthesized through a new protecting-group-free approach that simplifies their synthesis and furnishes them with diamino anchoring groups at each end (Scheme 1). Such terminal diamino groups enable the formation of stable molecular GNR junctions between two gold electrodes, which allows their single-molecule electrical investigation using the scanning tunneling microscope-based break-junction (STM-BJ) technique. Conductance measurements and theoretical calculations of this series of GNRs demonstrate long-range tunneling charge transport behavior along distances beyond 5.6 nm with a shallow length decay constant (0.068 Å^–1^) that is among the lowest for semiconducting GNRs.

## Results and Discussion

### Synthesis and Structural Characterization

The synthesis of **NR-6**, **NR-16**, and **NR-26** starts from 1,2,4,5-tetraaminobenzene **A**(66,75) and pyrenetetraone **B**(76) (Scheme 1). These building blocks have been equipped with tri-*iso*-butylsilyl and *tert*-butyl solubilizing groups, respectively, to ensure the solubility of the intermediates and the final GNRs. All of the details about the synthesis and characterization of the GNRs are given in the Supporting Information. To avoid the use of protective groups and reduce the number of synthetic steps, the cyclocondensation reactions were carried out with a 4-equivalent excess of either the tetraamine or the tetraone building block. By doing so, the formation of the double cyclocondensation adduct is favored versus the polymerization of the two components. The resulting dicyclocondensation adducts were isolated by column chromatography in all cases. **NR-6** (20%) was obtained by a double cyclocondensation of tetraone **B** with a 4-equivalent excess of tetraamine **A**. Then, **NR-16** (32%) was obtained by a cyclocondensation of **B** with a 4-equivalent excess of **NR-6**. Finally, **NR-26** was obtained in two steps by an iterative cyclocondensation between **B** and **NR-6**. First, **NR-6** underwent a double cyclocondensation with a 4-equivalent excess of tetraone **B** to yield tetraketone-terminated **NR-12** (61%), which was subsequently cyclocondensed with a 4-equivalent excess of **NR-6** to yield **NR-26** (29%).

**Figure 2:**
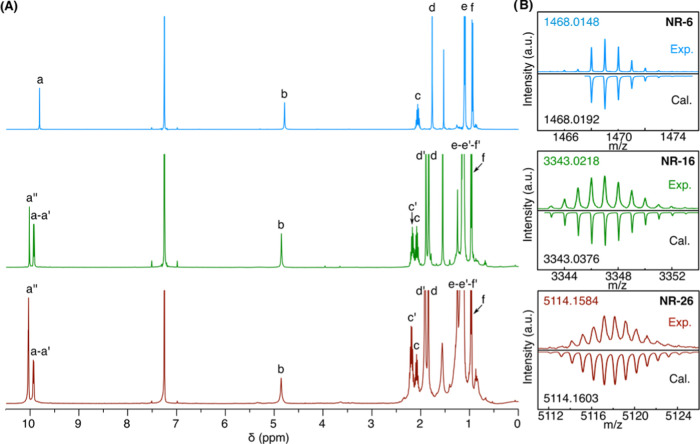
Structural characterization of the GNRs. (A) NMR spectra of **NR-6**, **NR-16**, and **NR-26** in CDCl_3_. (B) Mass spectra of **NR-6** ([M + H]^+^), **NR-16** ([M + Ag]^+^), and **NR-26** ([M + Ag]^+^).

The structures of **NR-6**, **NR-16**, and **NR-26** have been unambiguously confirmed by nuclear magnetic resonance (NMR) and high-resolution mass spectrometry (HRMS). The ^1^H NMR spectra of the different GNRs showed peaks and integrations that perfectly match with their structure, allowing us to establish unambiguously the length of the GNR (Figure 2A, the peak assignments correspond to the lettering shown in Scheme 1). For example, the signals of the terminal pyrene protons *a* and of the terminal tetramines *b* show a constant integration of 4 in all GNRs irrespectively of their length, whereas the integration sum of signals *a*′ and *a*′′ that correspond to the inner pyrene protons integrate 8 and 12 protons, respectively, for **NR-16** and **NR-26**. Furthermore, matrix-assisted laser desorption/ionization time-of-flight high-resolution mass spectrometry (MALDI-TOF HRMS) shows molecular ion peaks and isotopic distributions in agreement with the calculated ones (Figure 2B).

**Figure 3:**
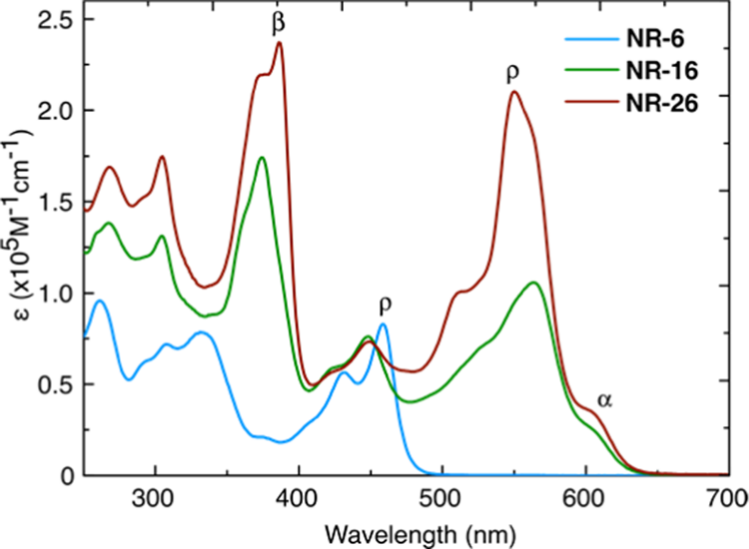
Electronic absorption spectra of **NR-6**, **NR-16**, and **NR-26** in CHCl_3_.

The optoelectronic and redox properties were investigated by UV–vis absorption and cyclic voltammetry, respectively. The UV–vis electronic absorption spectrum of **NR-6** (Figure 3) shows the typical β and ρ bands—at wavelengths ca. 326 and 462 nm, respectively—observed in dibenzohexacene and other pyrene-fused acene derivatives.^77−85^ The spectra of **NR-16** and **NR-26** show a similar absorption pattern in which the bands are red-shifted as a result of the extended π-system and also show a new band (α band) that dominates the spectra at low energies. This absorption pattern is again consistent with those observed for pyrene-pyrazinoquinoxaline GNRs and nanographenes.^56,57,67,86^ The energy of the ρ and α bands of **NR-16** and **NR-26** is practically the same, whereas the β band is red-shifted for **NR-26**. Furthermore, the molar absorptivities (ε) increase with the length of the GNRs, as illustrated by the ε values of the ρ band of **NR-6** (82,780 M^–1^ cm^–1^), **NR-16** (105,766 M^–1^ cm^–1^), and **NR-26** (210,266 M^–1^ cm^–1^). The optical highest occupied molecular orbital–least unoccupied molecular orbital (HOMO–LUMO) gap (*E*_gap_) was estimated from the onset of the longest wavelength transition. The optical *E*_gap_ values decrease from **NR-6** to **NR-16**, and then it saturates (2.61, 1.97, and 1.96 eV, respectively, for **NR-6**, **NR-16**, and **NR-26**). This trend is consistent with that observed on other pyrene-pyrazoquinoxaline molecular GNRs and nanographenes^56,57,67,86^ in which the absorption shifts rapidly, hence the bandgap, from 6 to 10 linearly fused rings and then for backbones >10 rings, the *E*_gap_ values saturate and remain almost invariable. The cyclic voltammograms carried out in solutions of *n*-Bu_4_NPF_6_ (0.1 M) in CH_2_Cl_2_ show irreversible reduction and oxidation waves in all cases (Figure S1). The potentials of the reduction waves appear at more cathodic potentials than those reported for similar molecular pyrene-pyrazinoquinoxaline GNRs,^56,67^ which is consistent with the presence of the four electron-donating amines. Similarly, to other molecular GNRs,^56,67^ the onset reduction potentials of the first wave appear at increasingly less cathodic potentials with increasing length (−1.33, – 1.01, and −0.98 V, respectively, for **NR-6**, **NR-16**, and **NR-26**). The presence of oxidation processes that had not been previously observed in similar pyrene-pyrazinoquinoxaline molecular GNRs^56,67^ was also attributed to the presence of the four amine substituents.

### Single-Molecule Charge Transport Studies

**Figure 4:**
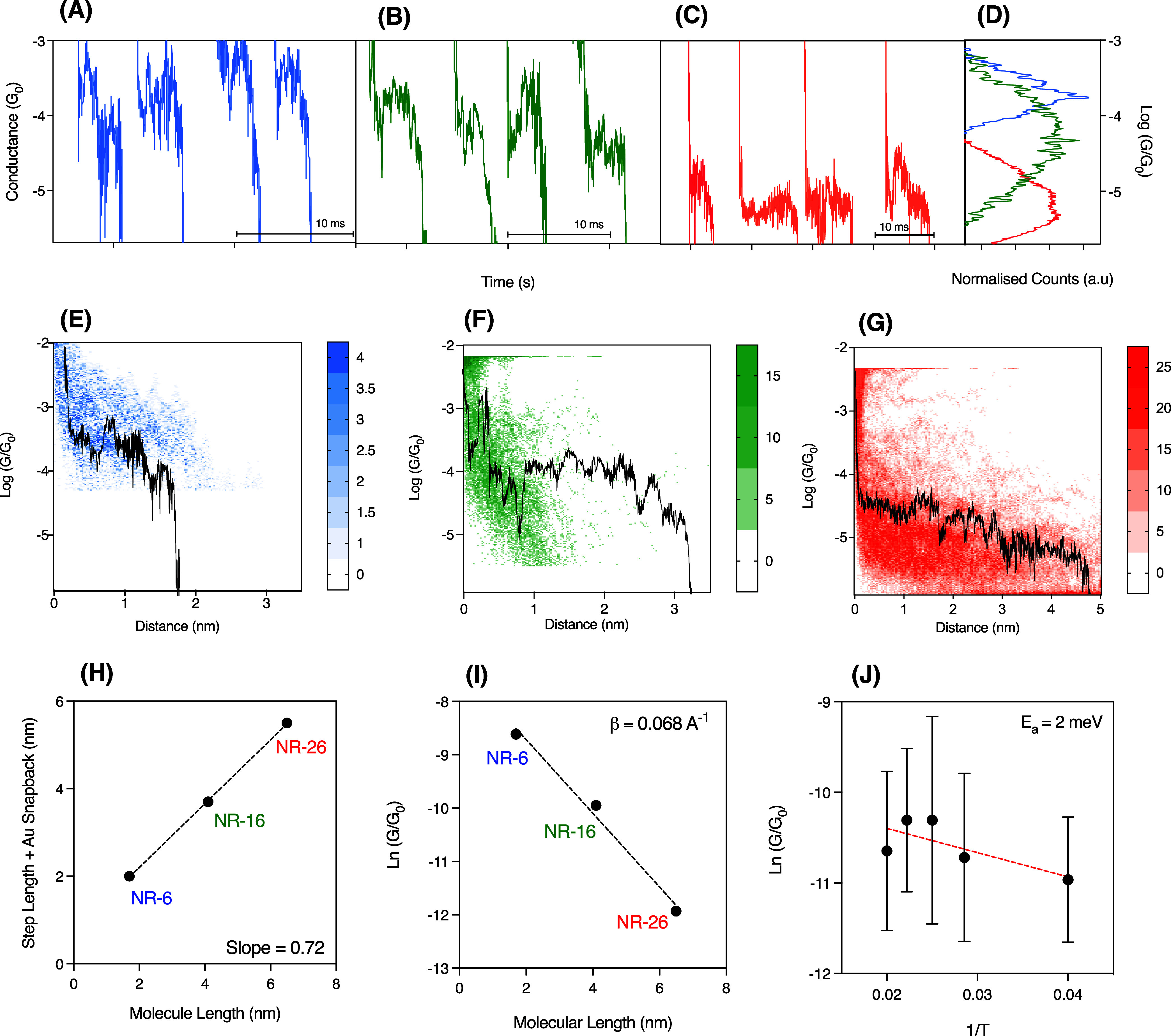
Charge transport results by STM break-junction. (A–C) Illustrative individual pulling traces for **NR-6**, **NR-16**, and **NR-26**, respectively. Panel (D) shows 1D conductance histograms built accumulating between 800 and 1000 traces displaying conductance plateau features. Peak maxima are extracted via a Gaussian fit (from the linear scale), representing the average conductance for each GNR. (E–G) 2D histograms accumulating hundreds of traces displaying current plateaus. Overlaid individual traces illustrate examples of long plateau lengths spanning the molecular length. (H) Correlation plot representing plateau length (including Au snapback) versus molecular length. (I) Semilog graph of GNR conductance versus molecular length. (J) Arrhenius representation of molecular conductance against 1/*T*. Applied bias voltage was 30 mV for **NR-6**, 200 mV for **NR-16** and 300 mV for **NR-26**.

Single-molecule charge transport characterization of each GNR was carried out in single-molecule junctions using the STM-BJ technique.^87,88^ In the STM-BJ approach, an Au STM tip is driven in and out of contact to/from an Au(111) substrate in a solution of the GNRs in 1,3,5-trimethylbenzene at very low concentrations (<10^–9^ M) to ensure an efficient anchoring of individual GNRs between the electrodes and to avoid molecular aggregation in solution. During the gap-opening process, individual GNRs can spontaneously bridge between both biased (a few mV) electrodes through the terminal *o*-diamine groups (Figure 1). Thousands of current versus gap separation curves (pulling traces) are recorded, from which 20–30% of those display current plateau features (Figure 4A–C) below the quantum conductance (*G*_0_ = 2*e*^2^*h*^–1^, where *e* is the electron charge and *h* is Planck’s constant). These plateau features indicate the successful formation of a molecular GNR junction and are accumulated to build conductance histograms whose maxima illustrate the most probable molecular junction (MJ) configuration (Figure 4D). Between 800 and 1000 pulling traces displaying plateaus (illustrative ones in Figure 4A–C) are selected using the same sorting algorithms and accumulated into 1D conductance histograms (Figure 4D), yielding average conductance values of 1.7 × 10^–4^*G*_0_ for **NR-6**, 4.6 × 10^–5^*G*_0_ for **NR-16**, and 6.5 × 10^–6^*G*_0_ for **NR-26**. To corroborate the formation of GNRs junctions, the same pulling traces displaying plateaus are accumulated in two-dimensional (2D) histograms, which visualize the conductance evolution as a function of the pulling process (Figure 4E–G). Average pulling lengths can be evaluated from the 2D histograms yielding 1.5, 3.2, and 5 nm for **NR-6**, **NR-16**, and **NR-26**, respectively, with the longest plateaus (see illustrative ones overlaid in Figure 4E–G) spanning nearly the full molecular length for each of the studied GNRs. Figure 4H shows an excellent correlation between the molecular length and the recorded longest plateau lengths in the break-junction experiment, adding the 0.5 nm of the Au snapback occurring during the Au–Au opening process.^17^ In the **NR-6** case, two different molecular configurations yielding two different conductance sets are observed (Figure S2), with the high conductance one corresponding to a molecular junction configuration likely probing a shorter electron pathway, as demonstrated by the much shorter plateau length associated with the high conductance feature (see illustrative overlaid trace in Figure S2). Both **NR-16** and **NR-26** display a single conductance feature with a number of traces spanning the full molecular length (Figure S2). The natural logarithm representation of the average conductance values associated with the molecule-long plateau features versus molecular length (Figure 4I) yields a linear behavior with a shallow slope, resulting in a length decay constant (β) of 0.068 Å^–1^. This β value is comparable to values reported for armchair GNRs.^70,73^ To deepen the charge transport mechanism for these systems, temperature dependence conductance measurements were carried out on the longest **NR-26**. Figure 4J shows the Arrhenius representation of the data around the temperature working range, yielding a negligible activation energy of ∼2 meV and supporting electron tunneling (*G* = *A* e^*–*ß*L*^, being *L* the molecular length) as the most plausible charge transport mechanism.^24−32^

### Theoretical Calculations

Computational calculations were carried out to support the mechanistic picture drawn above the GNR charge transport. Density functional theory (DFT) calculations (B3LYP-6-31G(d,p)) were used to get insight into the molecular structure of the GNRs. The models of **NR-6**, **NR-16**, and **NR-26** illustrate a virtually flat backbone with lengths of 1.7, 4.1, and 6.5 nm, respectively.

The DFT calculated (B3LYP/6-31G(d,p)) HOMO–LUMO gaps (3.18, 2.24, and 2.14 eV, respectively, for **NR-6**, **NR-16**, and **NR-26**) show a similar trend to the measured optical gaps. The calculations show three quasidegenerate HOMOs and two quasidegenerate LUMOs for **NR-6** (Figure S3), six quasidegenerate HOMOs and two quasidegenerate LUMOs for **NR-16** (Figure S4), and eight quasidegenerate HOMOs and four quasidegenerate LUMOs for **NR-26** (Figure S5).

In the case of **NR-6**, all quasidegenerate occupied and unoccupied orbitals spread longitudinally across the backbone, illustrating how the effect of the localization of Clar sextets on the off-linear pyrene rings in the linear conjugation is small (Figure S3). This is consistent with the high conductance values observed for **NR-6**.

In the case of **NR-16**, the electronic density in the quasidegenerate HOMO/HOMO–1 and LUMO/LUMO+1 couples is localized along nine central rings between the two terminal pyrenes, whereas in HOMO–2, HOMO–3, HOMO–4, and HOMO–5, the electronic density is localized at both ends of **NR-16** (Figure S4). Similarly, in the case of **NR-26**, the LUMO and LUMO+1 spread along nine central rings contained across the three central pyrenes, the HOMO–3 and HOMO–4 are localized on the central pyrazino-quinoxalines, the LUMO+2/LUMO+3 and HOMO/HOMO–1 couples are located in-between the two terminal pyrenes, and the HOMO–4 and HOMO–5 are localized at opposite ends of **NR-26** (Figure S5).

The localization of Clar sextets in the off-linear pyrene rings has a larger impact on the longitudinal conjugation of **NR-16** and **NR-26** that results in the compartmentalization of the orbitals in different segments along the GNR backbone, similar to what we have previously described in other pyrene-pyrazinoquinoxaline molecular GNRs and nanographenes.^56,57,67,86^ This is consistent with the fact that the dominant resonance structures of the GNRs are best represented by Clar’s sextet rule with biphenyl and anthracene groups on the pyrene and pyrazinoquinoxaline residues, respectively. This increasing level of compartmentalization of the electronic density with increasing lengths accounts for the decreasing conductance values. Remarkably, such compartmentalization does not interrupt the transport along the longer GNRs, as shown by the conductance values measured, respectively, for **NR-6** (1.7 × 10^–4^*G*_0_), **NR-16** (4.6 × 10^–5^*G*_0_), and **NR-26** (6.5 × 10^–6^*G*_0_), which are in the same order of magnitude typically observed in π-conjugated molecular wires.

**Figure 5:**
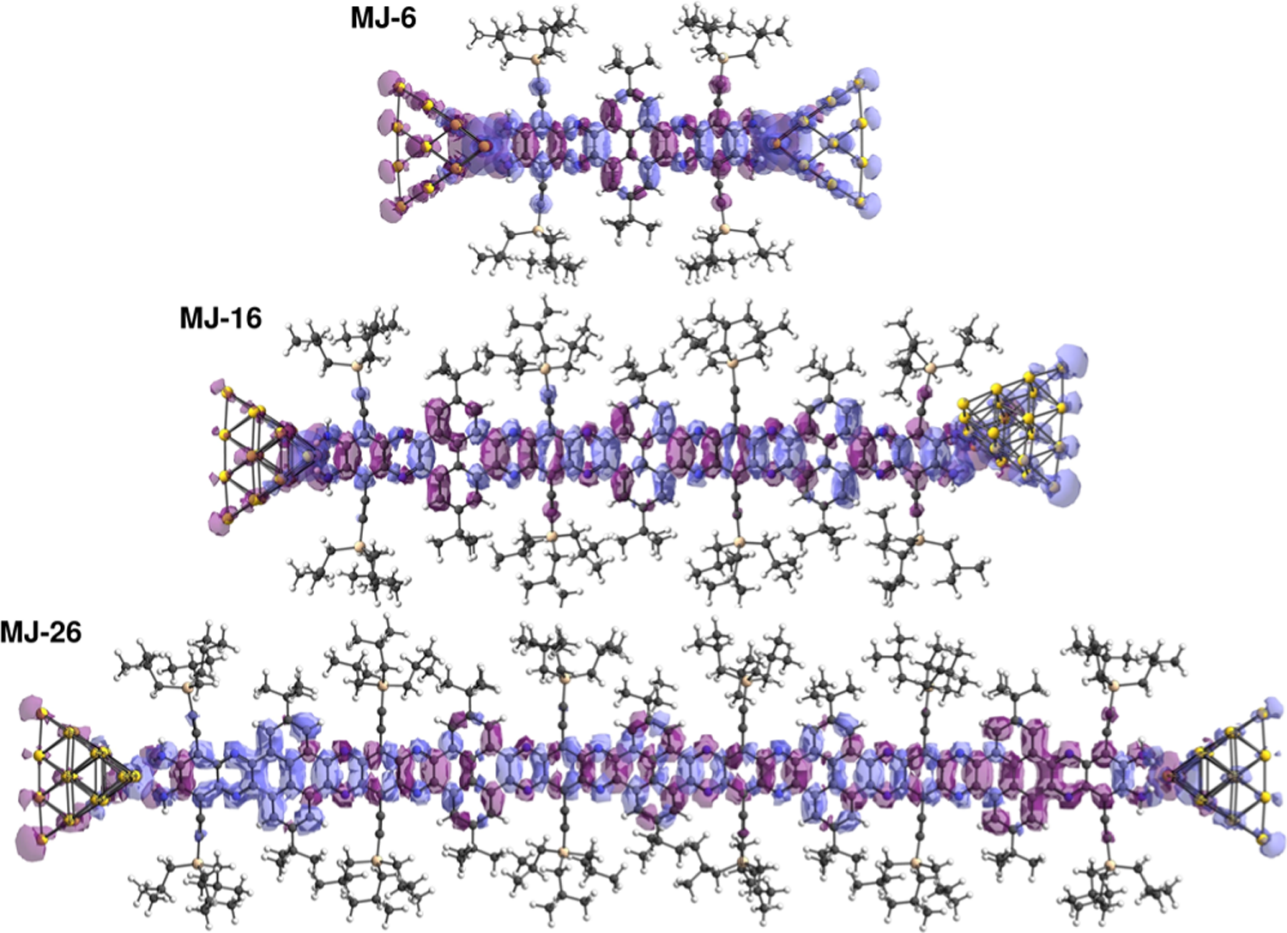
Sum of the individual channels, which contribute more than 75% to the total conductance trace of the molecular junctions **MJ-6**, **MJ-16**, and **MJ-26** and the gold tips under the bias voltage of 0.1 V calculated at the B3LYP-6-31G(d,p)/lanl2dz level of theory (e-function (purple) and hole function (blue)).

To understand how charge transport takes place across the GNR backbones, electron transport calculations in the frame of the time-energy uncertainty relation approach to molecular conductance were performed.^89^ Additionally, an MO analysis of the electron transport was carried out with the help of the electron deformation orbitals (EDOs).^90^ To get additional insight into the role played by the metal–molecule contacts in the electron transport efficiency, electron transport calculations were first performed for the individual GNRs (see below) and then for the molecular junction models, where the GNRs are complexed between metal contacts (Figure 5, where **MJ-6**, **MJ-16**, and **MJ-26** refer, respectively, to the molecular junctions (MJ) of **NR-6**, **NR-16**, and **NR-26**). Twenty gold atom pyramids represent the metal contacts. Despite the fact that there are some combinations and rearrangements of the GNRs and gold pyramids’ MOs, most of the π-MOs at the frontier region can be identified in the MO diagram of the molecular junction (Figures S6–S8) slightly displaced in position with respect to isolated GNRs (Figures S3–S5). The calculated conductance values decrease with the GNR length (Figure S9a) and fit an exponential decay, in agreement with the experimental trend. The theoretical β value (0.035 Å^–1^) of the molecular junction models is slightly lower but consistent with that obtained experimentally (Figure S9b). This is also consistent with the theoretical β value of the isolated molecular GNRs (0.047 Å^–1^), which indicates that the binding mode has a moderate influence on electron transport (Figure S9b).

As mentioned above, EDOs enable us to apply the MO theory to understand electronic transport at the molecular scale. Introducing the EDOs in the time-energy uncertainty relation approach to molecular conductance allows the construction of individual conducting channels built from mixing occupied and virtual orbital spaces induced by the electric perturbation. From the contributions of these channels to the total conductance, it is possible to identify the relevant orbitals that are involved in the electronic transport, giving rise also to a description of the process in terms of electron and hole density regions. In Figure 5, the sum of the individual channels that contribute more than 75% to the total conductance trace is represented. This is the distribution of electrons and holes (purple and blue, respectively), which describes the decrease of electron density (hole function) and the increase of electron density (electron function) within the MJ upon the application of the electric perturbation. In all GNRs, there is a large intercalation of electron and hole regions that increases with the length of the GNR. This distribution of the electron and hole functions bears some resemblance to the charge polarization in a dielectric material. It should be noted that the electron–hole boundaries are mainly located at the pyrene and pyrazine fragments. According to these representations, if the electron transport mechanism seems to be analogous for all the systems, then a length-dependent transition between mechanisms cannot be inferred. This is in accordance with the experimental conductance measurements at variable temperatures of **NR-26**.

Even if the mechanism seems to remain invariable, the length-dependent conductance decays can be understood in terms of the number of electron transport channels and of the frontier orbitals involved in the process and their distribution within the molecule. For instance, there are two main channels in **MJ-6** (Figure S10); the most important involves the HOMO, where the electron density is spread over the **NR-6** structure, and a set of quasidegenerate unoccupied orbitals mainly located at the gold tips and the outer rings of the GNR. This leads to a channel that spreads over the whole molecular junction. The second channel, however, arises from the mixing of both occupied and unoccupied orbitals located at the outer rings and the gold tips, leading to an electron transport channel without the contribution of the central pyrene ring. In **MJ-16** (Figure S11), the number of relevant channels increases to three, where the first two channels build up more than 60% of the total conductance. There are, however, significant differences between these channels and those from **MJ-6**. Thus, they are split and localized at different regions along the MJ (similarly to MOs), which, in combination, allow for an effective edge-to-edge charge transfer. The first channel, which is mainly distributed along the GNR edges, involves only the HOMO–2 and HOMO–6 orbitals, where the electronic density is concentrated at the edges and center of the GNR, respectively, and unoccupied orbitals are mainly located at outer rings and the gold tips. The second channel involves the HOMO–3 orbital located at the edges and gold tips and the LUMO+4 orbital mainly distributed in the central region of the GNR. Except for the central pyrene ring, this channel covers most of the backbone of the GNR. The remaining channel is mainly formed by HOMO–6 and LUMO orbitals, located mostly in the GNR’s center, leading to a channel that runs between the furthest pyrene rings. In **MJ-26** (Figure S12), the number of relevant channels increases to four, where the first three channels build up more than 60% of the total conductance (26, 23, and 18%, respectively). These channels involve occupied orbitals of the **NR-26** structure and a large set of unoccupied orbitals located at the tips and edges. Due to the different weights of these orbitals in each transport channel, there are noticeable differences between them. The first channel covers most of the backbone of the GNR, with the exception of the central pyrene and outer pyrazine fragments. The second channel is mainly located at the GNR’s edges, while the third channel again involves most of the MJ. The fourth channel, however, is the one that covers the largest surface of the GNR. These results are also consistent with those obtained for the isolated GNRs (Figures S13–S16).

## Conclusions

We synthesized a series of diamino-terminated molecular GNRs that reach 6.5 nm in length. The terminal diamines have proven to be optimal anchoring groups to prepare single-molecule Au-GNR-Au junctions by using an STM-BJ setup. This family of GNRs shows a mixed zigzag-cape edge structure that is known to compartmentalize the π-system, providing them with high stability. Conductance measurements show that this compartmentalization does not interrupt the longitudinal transport along the GNRs, which deliver conductance values in the same order of magnitude as those typically observed in π-conjugated molecular wires, giving rise to a low length decay constant (0.068 Å^–1^) that is among the lowest measured for semiconducting graphene GNRs.^70,73^ Variable temperature conductance measurements, together with computational calculations, suggest that charge transport is dominated by electron tunneling within the studied length scales. The conductance decay with length can be understood with regard to the number of conducting channels involved in the process and its distribution within the molecule. Overall, we reveal a comprehensive picture of the charge transport phenomena that arise in long Au-GNR-Au junctions. The study of electron transport through such junctions shines light on the fundamental charge transport properties of GNRs and opens up prospects for the study of nanoscale charge transport for other types of ex situ-synthesized GNRs.

## Supporting Information Available

Supporting figures, additional experimental details, and materials and methods, including NMR and MS spectra for all compounds (PDF)

## Abbreviations

GNRs: graphene nanoribbons
NR: nanoribbon
STM-BJ: scanning tunneling microscope-based break-junction
NMR: nuclear magnetic resonance
HRMS: high-resolution mass spectrometry
MALDI-TOF HRMS: matrix-assisted laser desorption/ionization time-of-flight high-resolution mass spectrometry
β: length decay constant
MJ: molecular junction
DFT: density functional theory
EDOs: electron deformation orbitals
