# Supplementary material for: Molecular Graphene Nanoribbon Junctions
Source: J Am Chem Soc. 2024 Feb 2;146(6):3963–73. doi: 10.1021/jacs.3c11340 (PMC10870704; doi:10.1021/jacs.3c11340)
Supplement: Supplementary file 1 — ja3c11340_si_001.pdf [file ja3c11340_si_001.pdf]

Supporting Information

Molecular Graphene Nanoribbon Junctions

Mauro Marongiu,[1] Tracy Ha,[2] Sara Gil-Guerrero,[3] Kavita Garg,[2] Marcos Mandado,[4] Manuel Melle-Franco,[3]* Ismael Diez-Perez,[2]* and Aurelio Mateo-Alonso[1,5]*

[1] *POLYMAT, University of the Basque Country UPV/EHU, Avenida de Tolosa 72, 20018 Donostia-San Sebastian, Spain. E-mail: amateo@polymat.eu*

[2] *Department of Chemistry, Faculty of Natural & Mathematical Sciences, King's College London, Britannia House, 7 Trinity Street, London SE1 1DB, United Kingdom. E-Mail: ismael.diez_perez@kcl.ac.uk*

[3] *CICECO - Aveiro Institute of Materials, Department of Chemistry, University of Aveiro, 3810-193 Aveiro, Portugal. E-mail: manuelmelle.research@gmail.com*

[4] *Department of Physical Chemistry, University of Vigo, Lagoas-Marcosende s/n, 36310 Vigo, Spain*

[5] *Ikerbasque, Basque Foundation for Science, 48009 Bilbao, Spain*

Supporting Figures

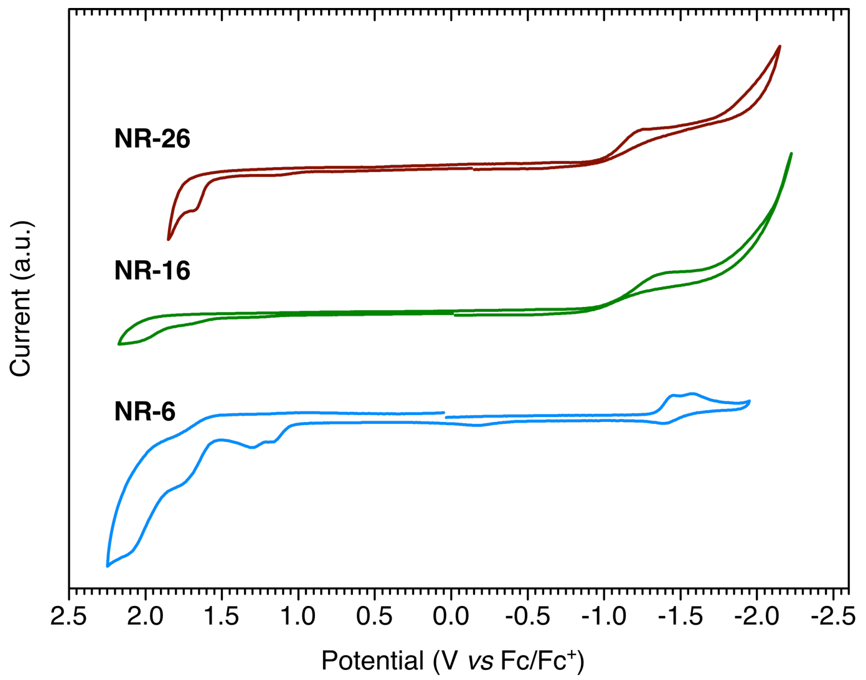

Figure S1. Cyclic voltammograms of **NR-6**, **NR-16** and **NR-26** (100 mV/s) in a solution *n*-Bu_4NPF_6 (0.1 M) in CH_2Cl_2 under argon and the ferrocene/ferrocenium (Fc/Fc^+) redox couple as internal standard.

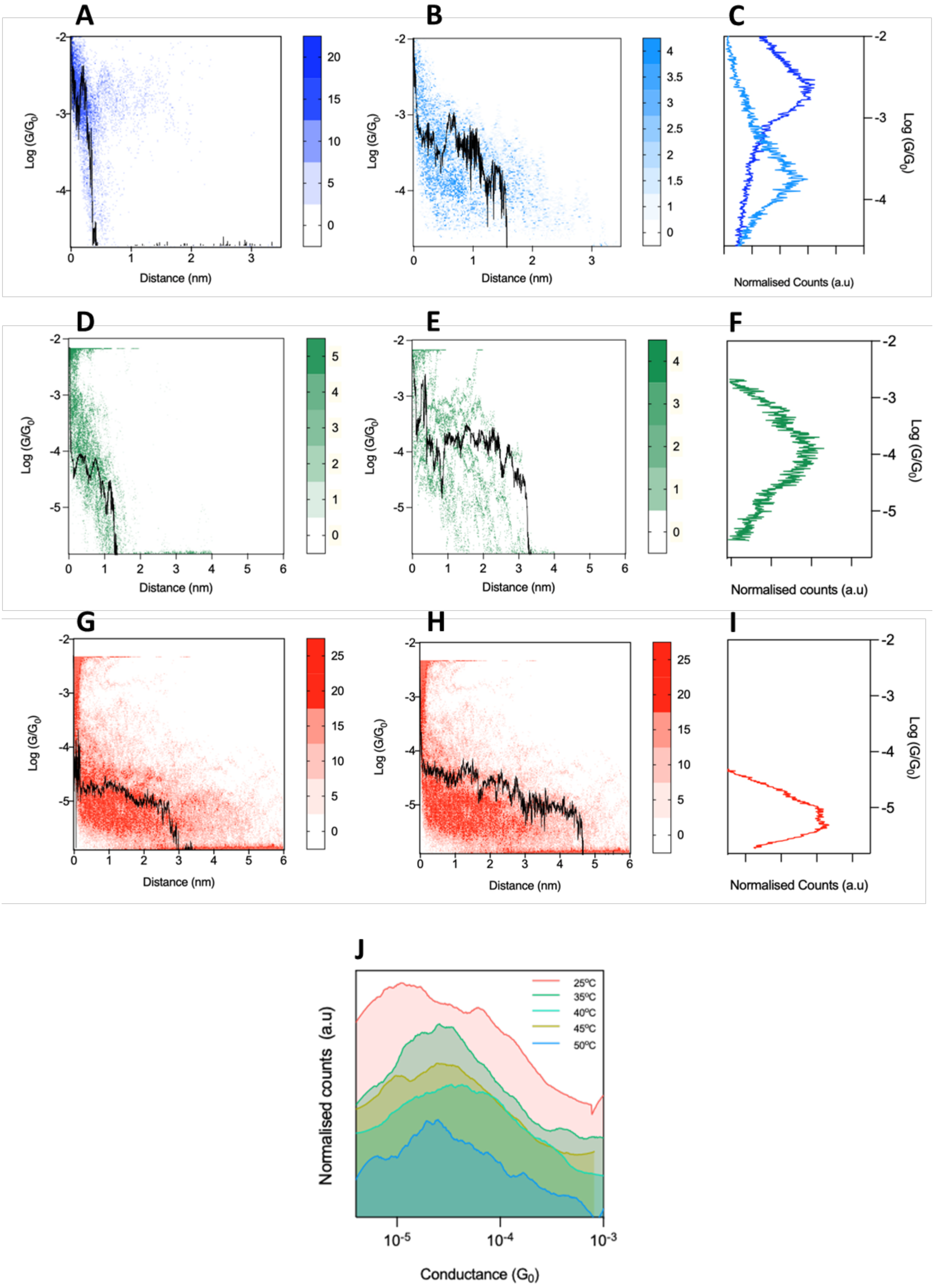

Figure S2. (A-C) 1D and 2D histograms for **NR-6** showing two (high and low) conductance features. (D-F) and (G-I) 1D and 2D histograms for **NR-16** and **NR-26**, respectively, displaying a unique conductance feature which is independent on plateau length. (J) 1D conductance histograms of **NR-26** at different temperatures.

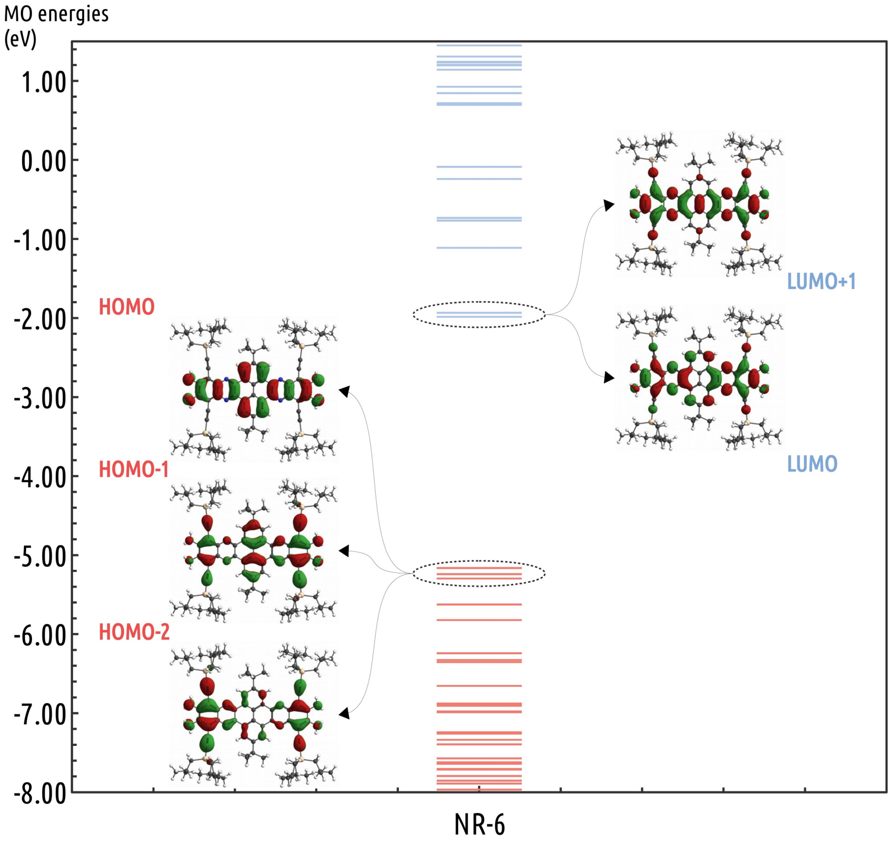

Figure S3. Kohn–Sham orbital eigenvalues of **NR-6** within an energy window spanning from −8 to 1 eV calculated with the B3LYP-6-31G(d,p) level of theory, and wavefunction densities of the degenerate frontier orbitals (isosurface=0.02).

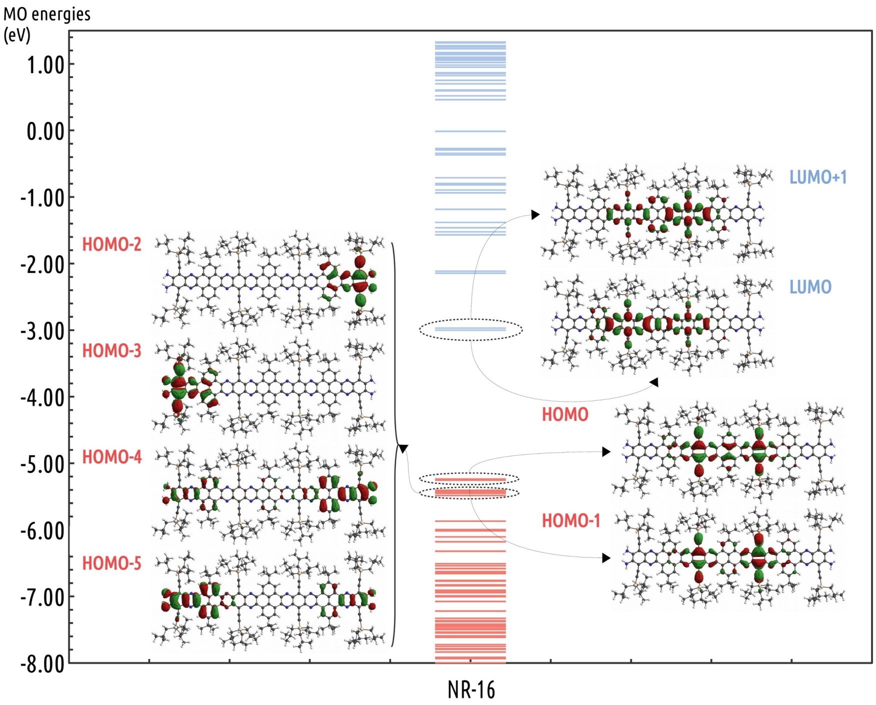

Figure S4. Kohn–Sham orbital eigenvalues of **NR-16** within an energy window spanning from −8 to 1 eV calculated with the B3LYP-6-31G(d,p) level of theory, and wavefunction densities of the degenerate frontier orbitals (isosurface=0.02).

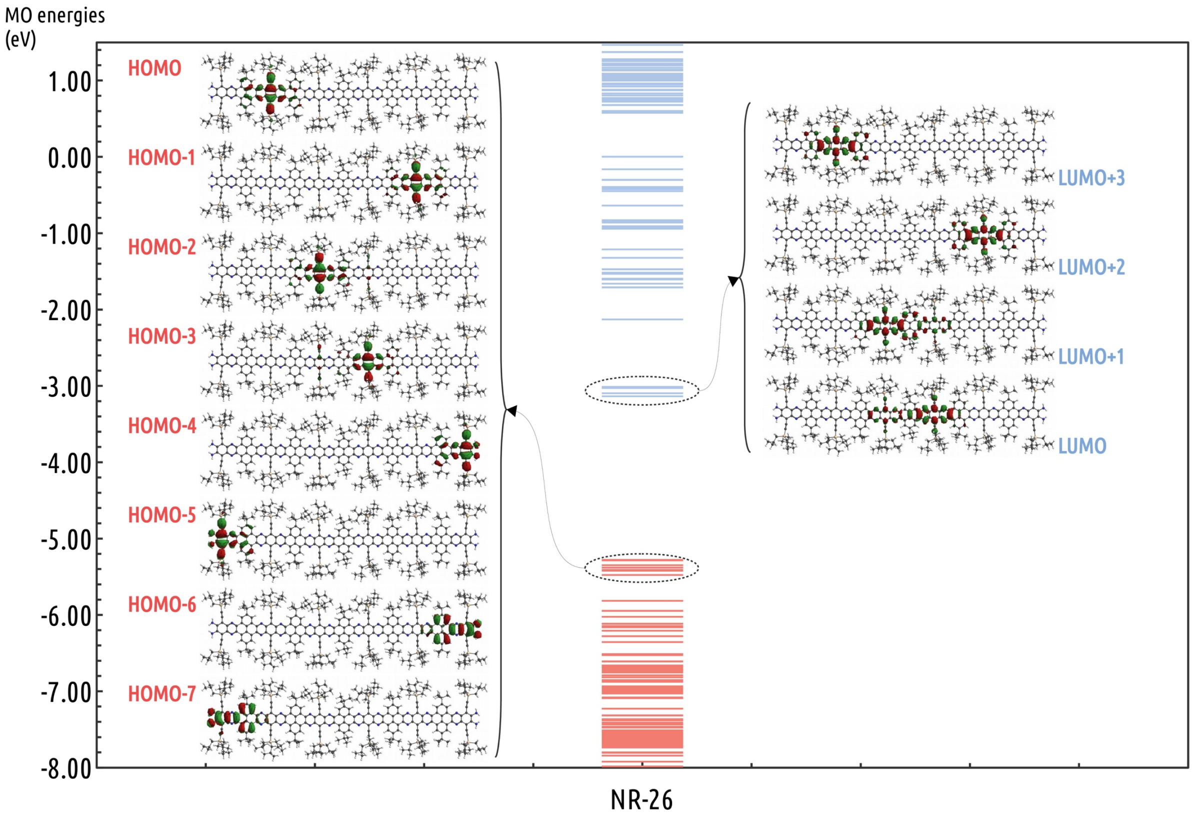

Figure S5. Kohn–Sham orbital eigenvalues of **NR-26** within an energy window spanning from −8 to 1 eV calculated with the B3LYP-6-31G(d,p) level of theory, and wavefunction densities of the degenerate frontier orbitals (isosurface=0.02).

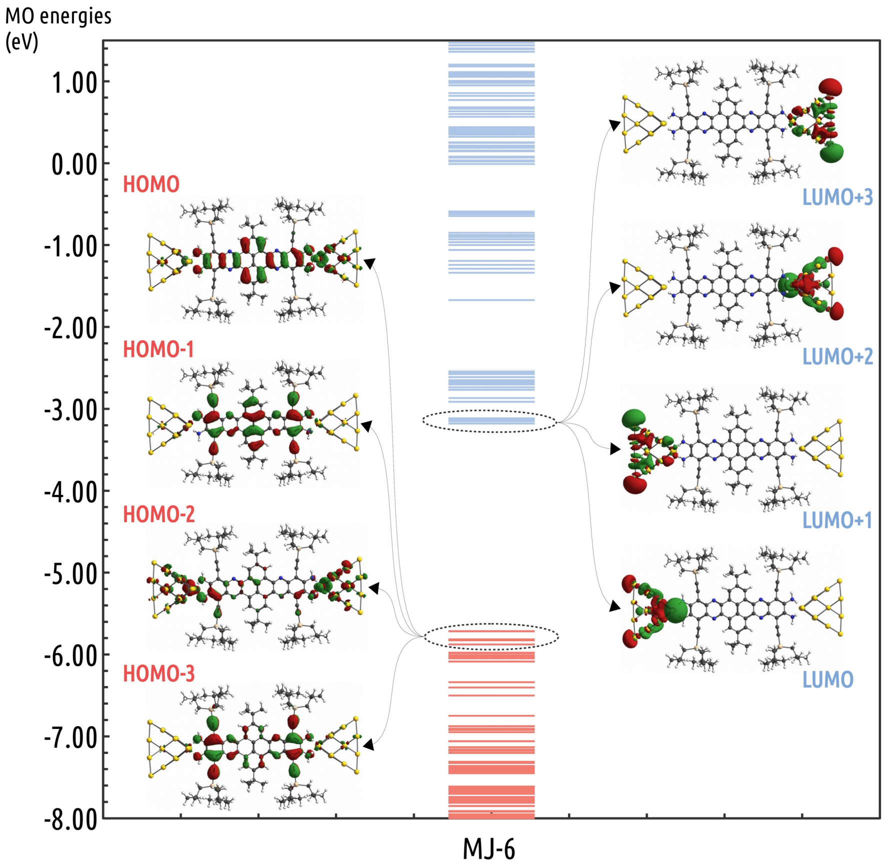

Figure S6. Kohn–Sham orbital eigenvalues of **MJ-6** within an energy window spanning from −8 to 1 eV calculated with the B3LYP-6-31G(d,p)/lanl2dz level of theory, and wavefunction densities of the degenerate frontier orbitals (isosurface=0.02).

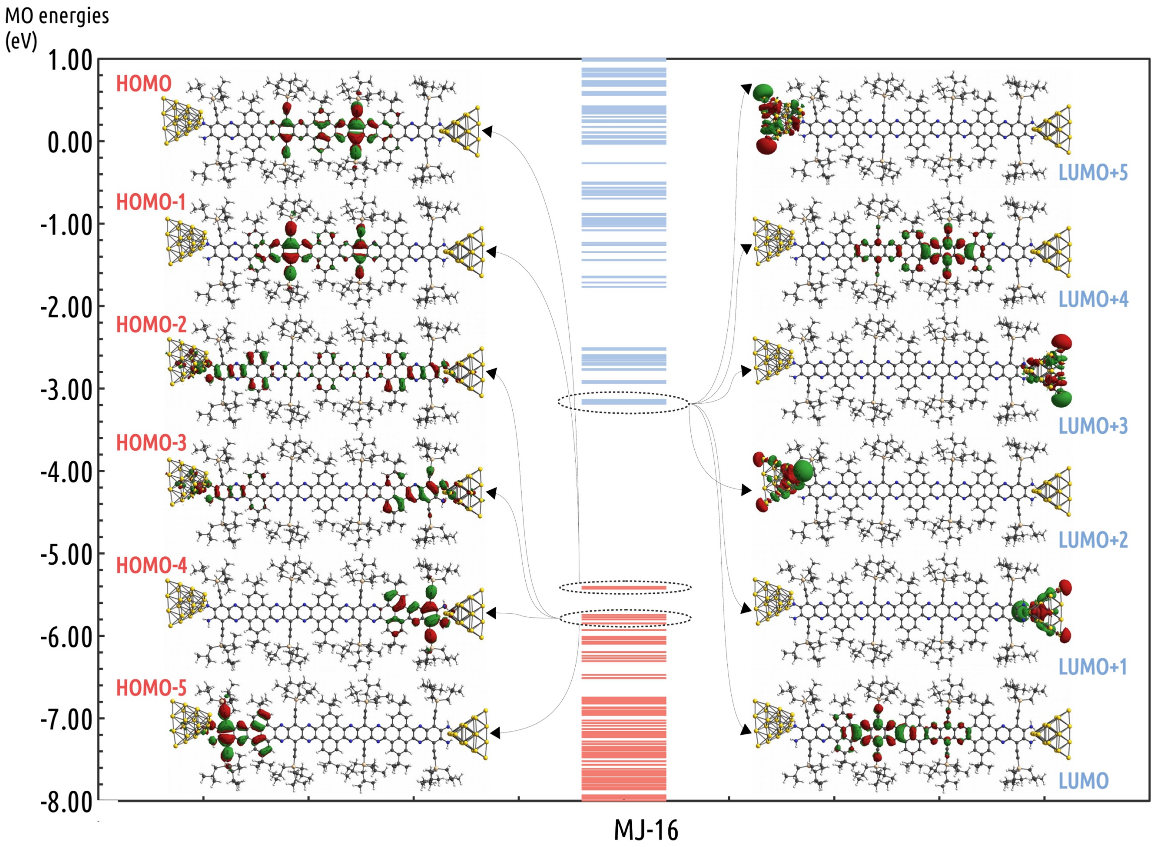

Figure S7. Kohn–Sham orbital eigenvalues of **MJ-16** within an energy window spanning from −8 to 1 eV calculated with the B3LYP-6-31G(d,p)/lanl2dz level of theory, and wavefunction densities of the degenerate frontier orbitals (isosurface=0.02).

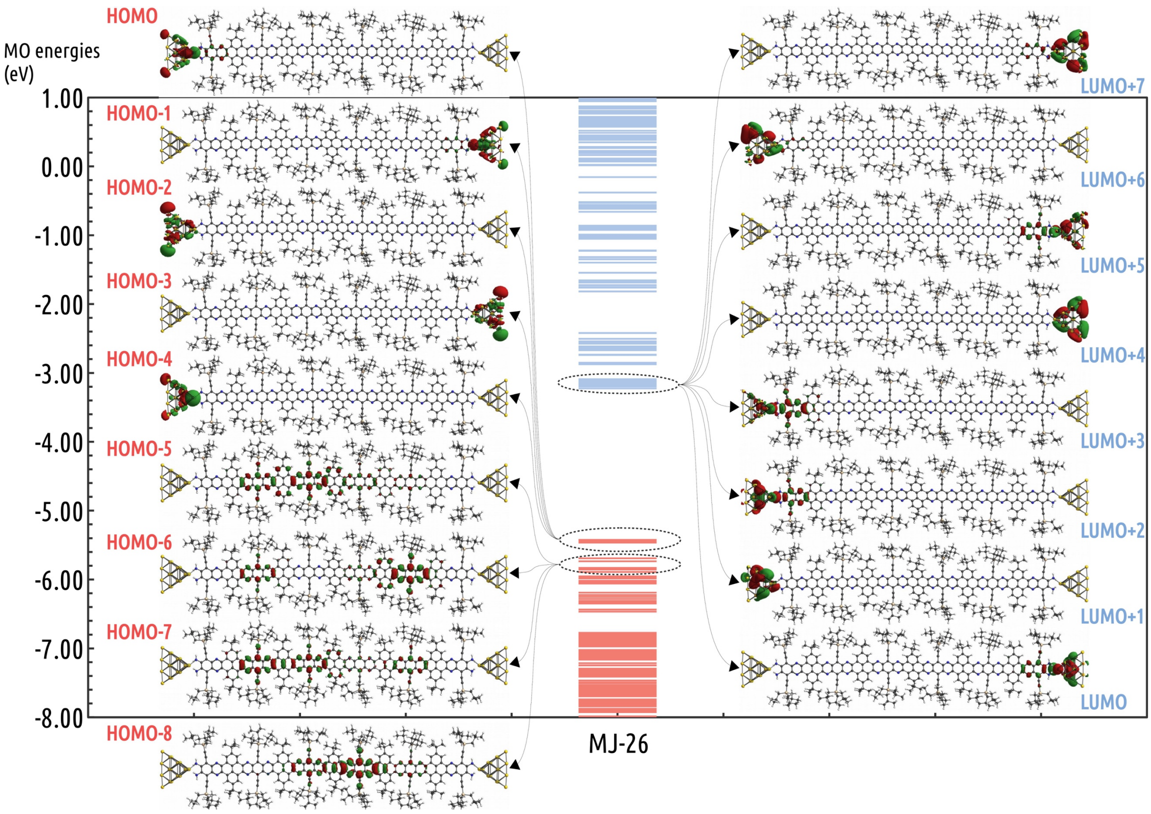

Figure S8. Kohn–Sham orbital eigenvalues of **MJ-26** within an energy window spanning from −8 to 1 eV calculated with the B3LYP-6-31G(d,p)/lanl2dz level of theory, and wavefunction densities of the degenerate frontier orbitals (isosurface=0.02).

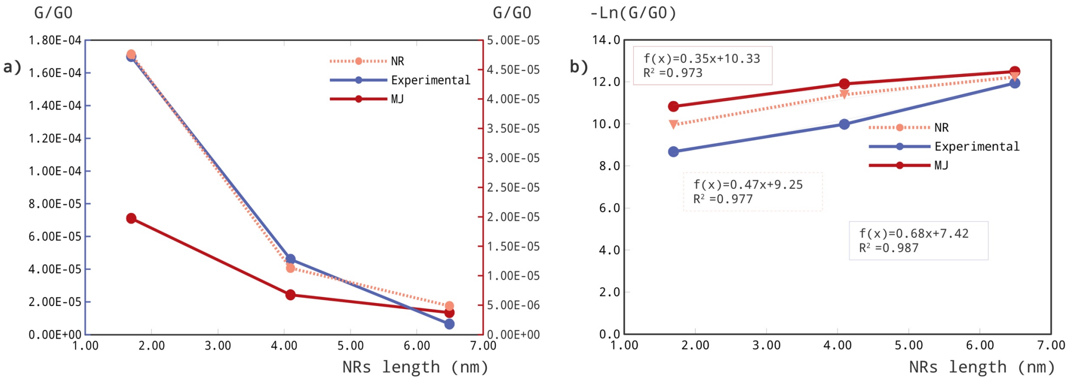

Figure S9. A) Comparison between the experimental electrical conductance (G/G_0) for the **MJ-6, MJ-16** and **MJ-26** molecular junctions (blue trace) and the calculated G/G_0 within the EDOs formalism under a bias voltage of 0.1V for the **NR-6**, **NR-16** and **NR-26** nanoribbons (dotted trace) and for the **MJ-6, MJ-16** and **MJ-26** molecular junctions (red trace) and B) their corresponding ß values.

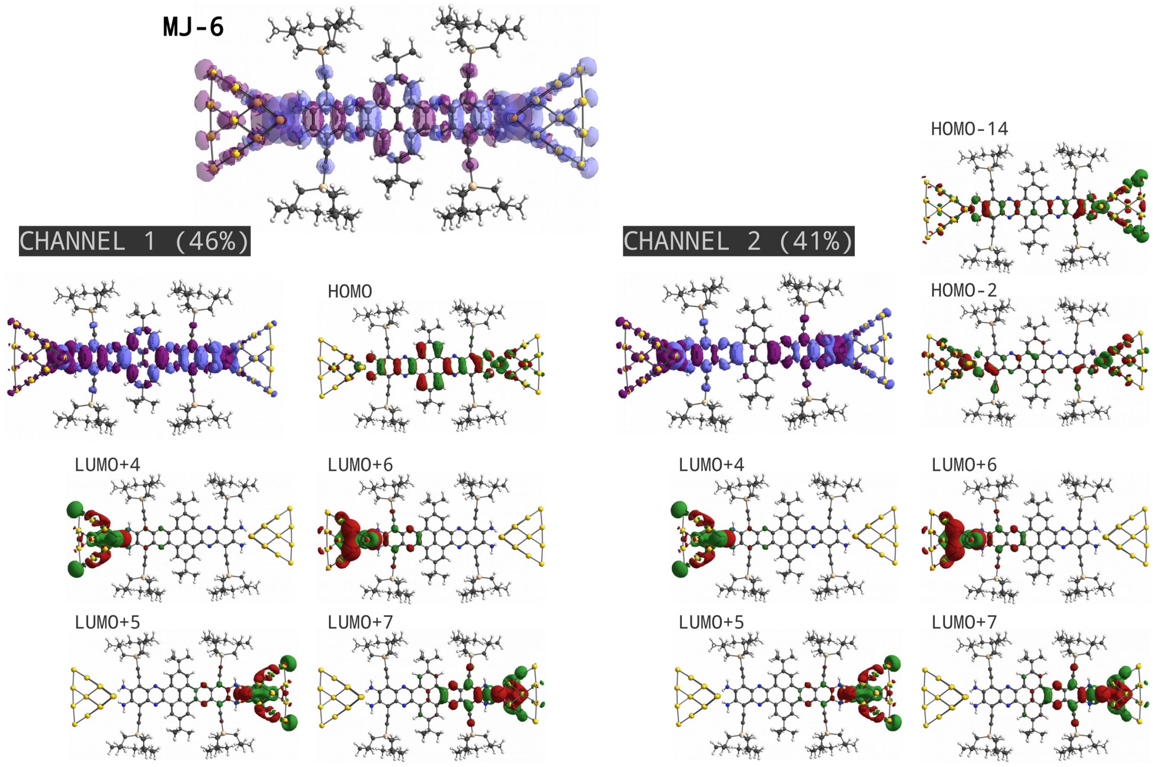

Figure S10. Single electron transport channels sorted by their contribution, together with the constituent occupied and unoccupied MOs of **MJ-6**.

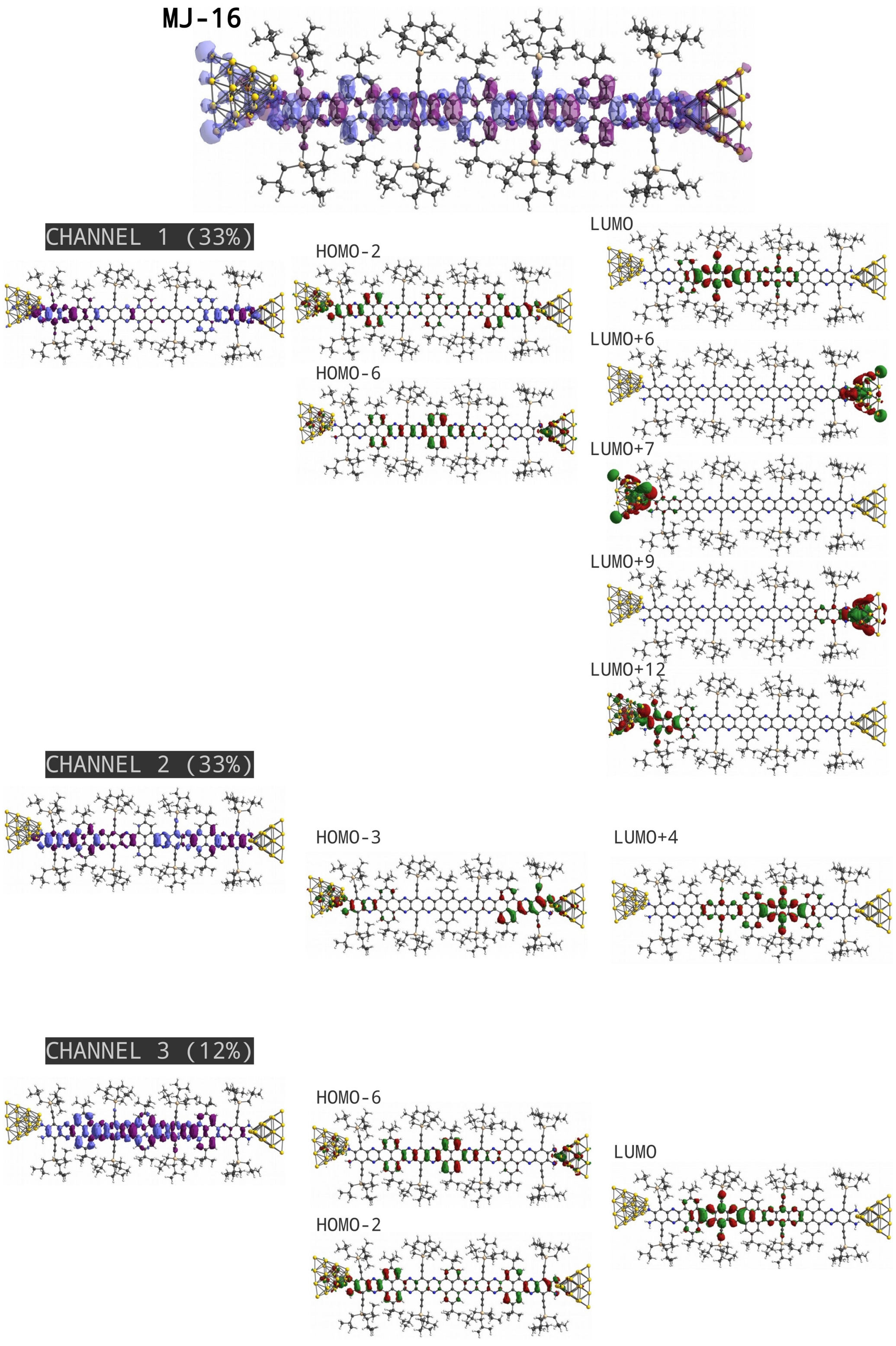

Figure S11. Single electron transport channels sorted by their contribution, together with the constituent occupied and unoccupied MOs of **MJ-16**.

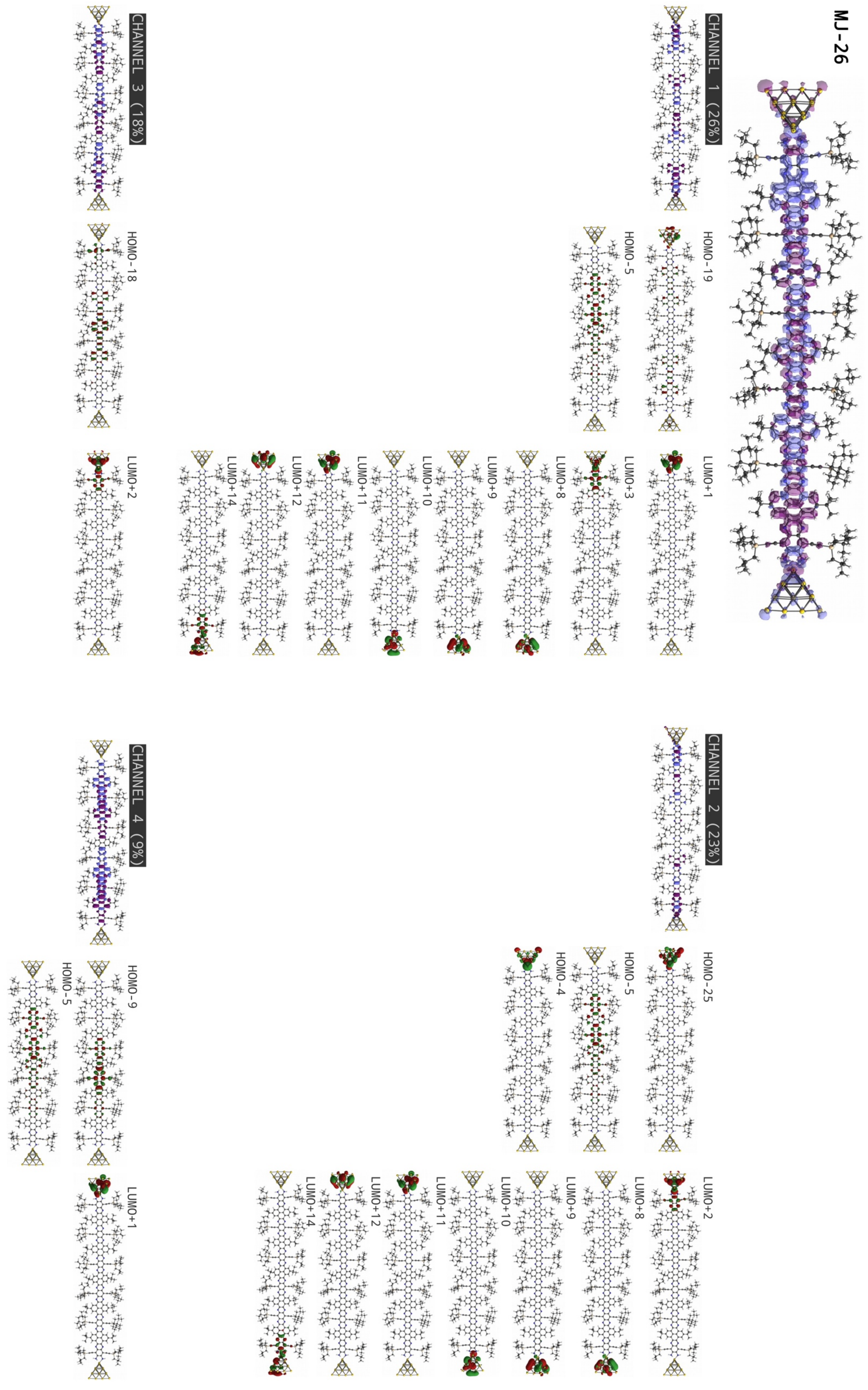

Figure S12. Single electron transport channels sorted by their contribution, together with the constituent occupied and unoccupied MOs of **MJ-26**.

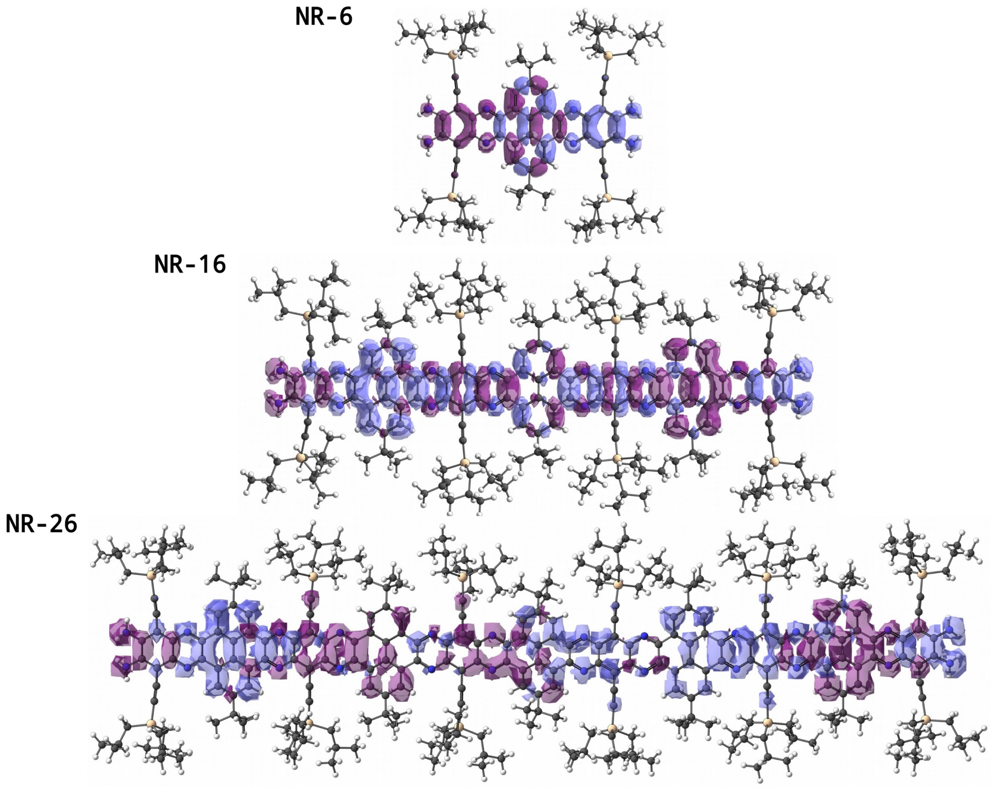

Figure S13. Sum of the individual channels which contribute more than 85% to the total conductance trace of **NR-6**, **NR-16** and **NR-26** under a bias voltage of 0.1V, calculated with B3LYP-6-31G(d,p) level of theory. The electron functions are rendered in purple while the hole function is rendered in blue. The analysis of the electron transport within the MJ set-up is presented in the main body of this article. Herein, a detailed analysis of the GNRs without electrodes is given for comparison.

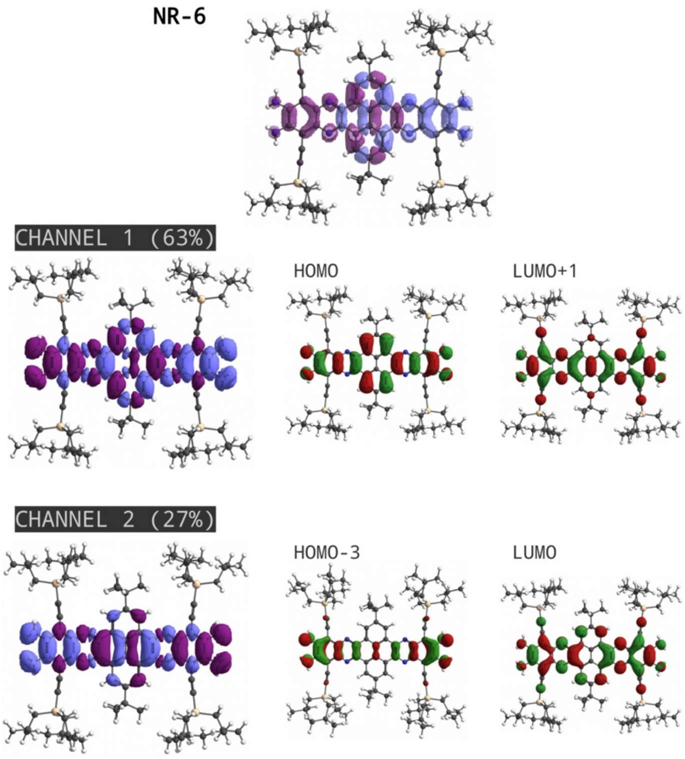

Figure S14. Single electron transport channels sorted by their contribution, together with the constituent occupied and unoccupied MOs in **NR-6**. For **NR-6** there are two main channels that involve HOMO and LUMO+1, and HOMO-3 and LUMO respectively. In both cases, the channel covers the entire structure's backbone.

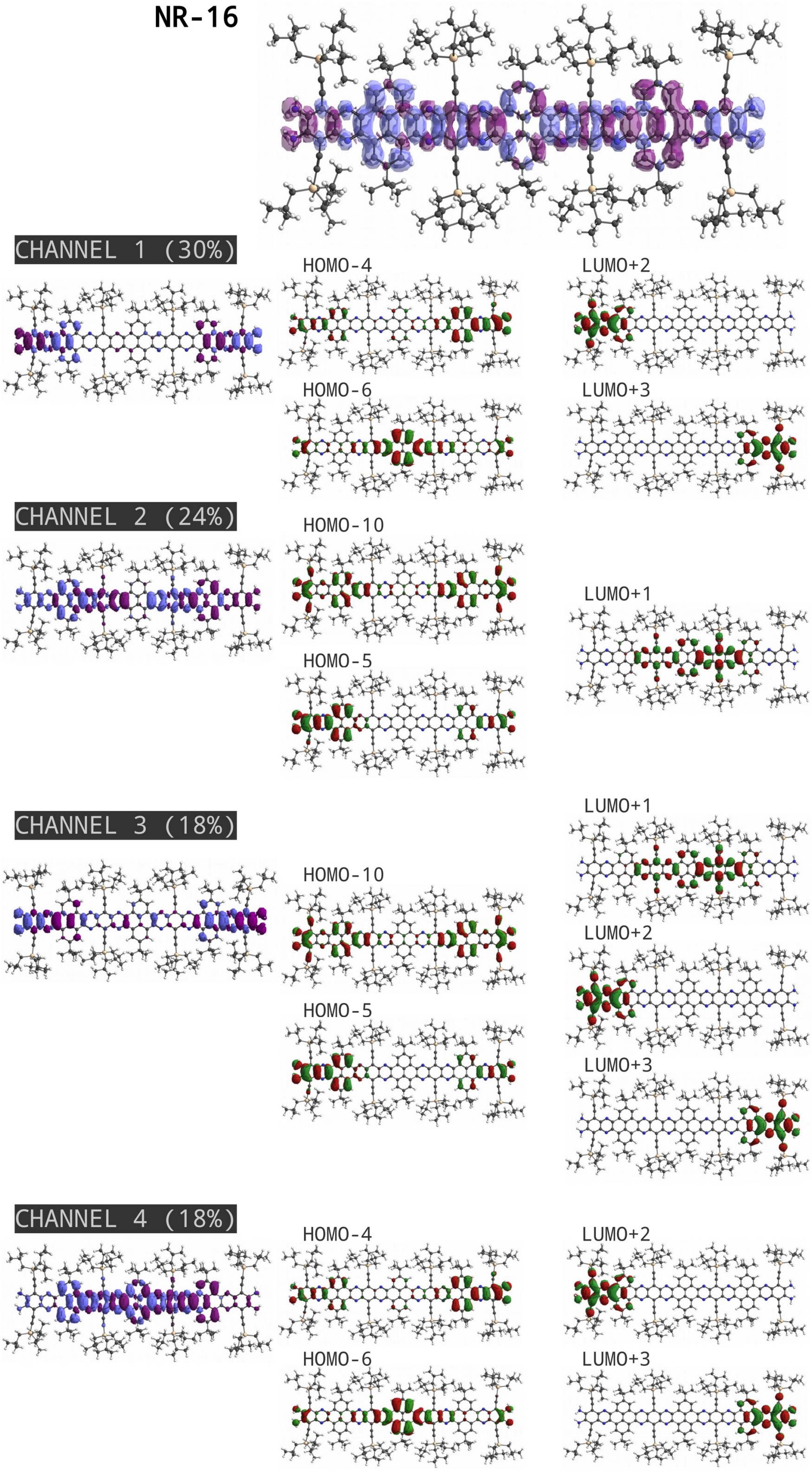

Figure S15. Single electron transport channels sorted by their contribution, together with the constituent occupied and unoccupied MOs in **NR-16**. For **NR-16** the number of relevant channels increases to four and there is a significant localization of these channels in different regions of the structure. While channels 1 and 3 are mainly located at the edges of the NR, channels 2 and 4 comprise only the central fragment.

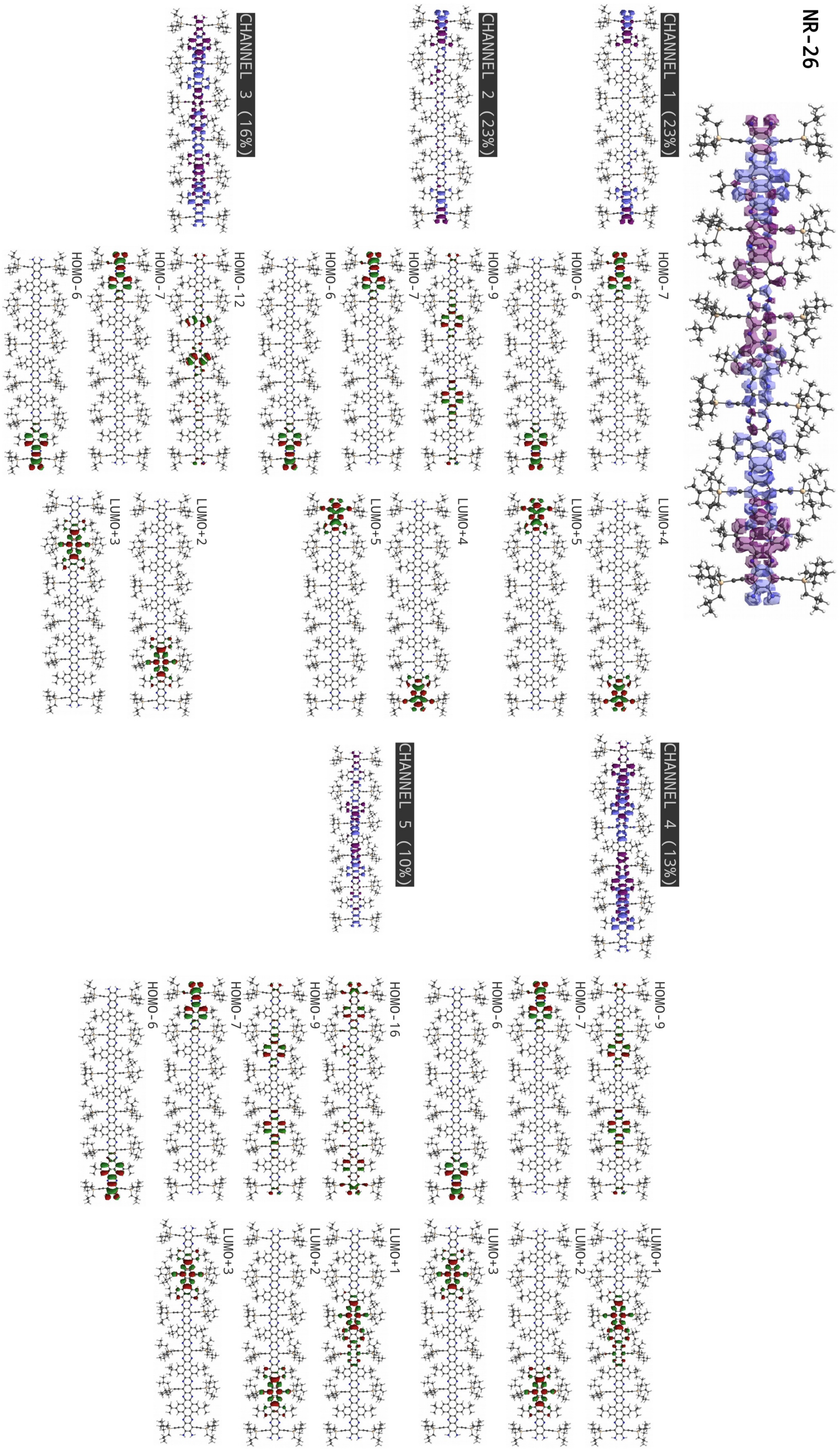

Figure S16. Single electron transport channels sorted by its contribution, together with the constituent occupied and unoccupied MOs in **NR-26**. For **NR-26** the two main transport channels (46%) are localized at the outer rings of the structure, while the third and fourth channels cover most of the NR's backbone and the fifth channel is confined to its central region.

Materials and Methods

Reagents. All the commercial reagents utilized in the synthesis were used as received. 3,6-Bis((triisobutylsilyl)ethynyl)benzene-1,2,4,5-tetraamine,[1, 2] and pyrene-4,5,9,10-tetraketone[3] were synthesized following literature procedures.

Synthesis. All the reactions were performed in an oven-dried round-bottom flask, Schlenk tube or reaction vial. The required high temperature for the reactions was achieved by the use of an oil-bath or an aluminium heating block, unless otherwise noted. The reaction progress was regularly monitored by thin-layer chromatography on TLC plates for which visualization was realized either by visual observation with naked eye or by irradiation with UV lamp.

Purification. The purification of the products was performed by silica-gel column chromatography under ambient conditions. The sorbent for the column chromatography (silica gel 60, 0.04–0.06 mm, 230–400 mesh) and the TLC plates were purchased from commercial suppliers. The monitoring of the purification process was performed by thin-layer chromatography on TLC plates.

Characterization. The NMR spectra were recorded with 400 or 500 MHz pulsed Fourier transform NMR spectrometer in deuterated solvents at room temperature. The chemical shift values are given in ppm and *J* values in Hz. High-resolution mass spectra of all the compounds were recorded by Dr. Javier Calvo on UltrafleXtreme III MALDI tandem mass spectrometer (Bruker) in reflector acquisition operation mode and the samples were prepared in THF.

Absorption and emission spectroscopy. The absorption spectra were recorded with a double beam UV/VIS/NIR spectrophotometer (PerkinElmer – Lambda 950) and the emission spectra were collected on a fluorescence spectrometer (PerkinElmer – LS 55). Fluorescence quantum yields were determined by the comparative method using cresyl violet perchlorate3 (Φf = 0.54 in methanol) as a standard.

Cyclic voltammetry. Electrochemical behavior of the compounds was studied using cyclic voltammetry in a three-electrode single-compartment cell consisting of a glassy carbon working electrode, silver wire as the reference electrode, and a platinum wire as the counter electrode with ferrocene (Fc) as an internal reference. The cell was connected to the computer controlled potentiostat (Princeton Applied Research - PARSTAT 2273). The measurements were carried out under Ar atmosphere in anhydrous CH_2Cl_2 using tetrabutylammonium hexafluorophosphate (0.1 M) as the supporting electrolyte. The concentration of molecular GNRs was 0.5 mM.

STM Break junction. Details about the STM-break junction technique have been published elsewhere.[4] All the conductance measurements were carried out with a mechanically and electronically isolated Bruker STM microscope head controlled by a Nanoscope V electronics (Bruker) and using a homemade PTFE STM cell. Data captures were acquired using a NI-DAQmx/BNC-2110 National Instruments (LabVIEW data acquisition System) and analyzed with LabVIEW code. The procedure of a typical break-junction experiment is based on bringing the STM tip to tunneling distance over a flat clean Au (111) surface area as a first step. The STM feedback is then turned off and the tip is driven into and out of contact with the substrate at a speed of 1-2V/s. This 2-points feedback loop is used to capture thousands of current decays (4000-5000). Single molecule conductance (G) was determined using the expression $G=I_{step}/U_{BIAS}$, where I is the current and U is the voltage

difference between the two junction electrodes. The current decays are accumulated to semi-logarithmic conductance histograms. The observed plateaus in the individual current decays result in the observed peaks in the conductance histograms and provide an averaged value of the single-molecule conductance. Transient curves that are either noisy or that showed smooth exponential decay because of the absence of molecular bridge formation were rejected when building the histograms using an automatic selection procedure driven by a code written in LabVIEW. The histograms were compiled by applying the same automated selection criteria to each set of the recorded decay curves. The selection procedure allows current traces showing counts exceeding a defined threshold to be added to the conductance histogram. The percentage decay curves that showed clear molecular steps were typically 20–30% and were all selected to build the histograms.[5-7] This selection process made peaks in the 1D conductance histograms more prominent (or regions with a higher number of counts in the 2D maps) above the tunneling background and also allowed a quantitative measure of the yield of molecular junction formation in all conductance traces.
The 2D logarithmic maps were performed by a code written in LabVIEW. The 2D maps are built from the captured current decays, accumulating the collected counts in the selected conductance sections of the whole measured conductance range.[8] The same selection criteria used to build the analogous 1D histograms were employed.

Computational Details. The geometry optimizations of the GNRs were done with the Gaussian09 software at the B3LYP-6-31G(d,p) level of theory. A pyramid of twenty gold atoms was employed to simulate the electrodes; its geometry was fully optimized at the B3PW91/lanl2dz level of theory including pseudopotentials to describe the core electrons. Then, the Molecular Junctions (MJs) were constructed with the tip of the gold pyramids pointing at the lone pair of the amino groups located at the GNR edges. The interaction between the tip and the nitrogen lone pair was mapped so that the minimum energy orientation and distance were chosen for the electron transport calculations. Finally, single point calculations were carried out at the B3LYP-6-31G(d,p)/lanl2dz level of theory with the MJ subjected to an external electric potential of 0.1V to obtain the corresponding conductance (G) values by means of the Electron Deformation Orbital (EDO) approach. EDOs allow understanding the electron transport process within the framework of the Molecular Orbital (MO) theory.

Synthesis

Synthesis of Compound NR-6:

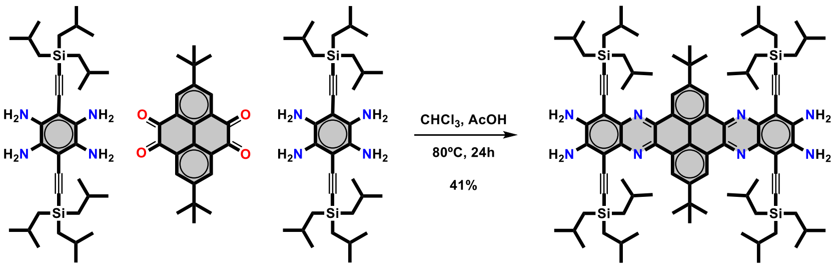

A B A NR-6

An oven dried sealable vial was charged with 3,6-bis((triisobutylsilyl)ethynyl)benzene-1,2,4,5-tetraamine **A** (0.758 g, 1.300 mmol, 4.0 eq.) and pyrene-4,5,9,10-tetraketones **B** (0.122 g, 0.325 mmol, 1.0 eq.). Subsequently, anhydrous chloroform (4 mL) and glacial acetic acid (1 mL) were added and the vial was sealed with a PTFE/Silicone septum. The reaction was left to stir 24 hours at 80°C. After being cooled to room temperature, the crude product was extracted with dichloromethane (3 x 50 mL) and the organic layer was washed with water. The combined organic phase was concentrated and the residue was purified by silica-gel column chromatography eluting with hexane – dichloromethane (4:1) to afford the product **NR-6** (0.098 g, 20%).

^{1}H NMR (400 MHz, $CDCl_3$) δ 9.82 (s, 1H), 4.80 (s, 2H), 2.07 (m, *J* = 13.3, 6.7 Hz, 3H), 1.77 (s, 5H), 1.11 (d, *J* = 6.6 Hz, 20H), 0.95 (d, *J* = 6.9 Hz, 6H).

^{13}C NMR (126 MHz, $CDCl_3$) δ 149.84, 142.88, 140.64, 139.14, 129.81, 125.04, 123.27, 106.61, 104.17, 101.06, 35.94, 32.47, 26.71, 25.48, 25.46.

HRMS (MALDI-TOF) Calculated for $C_{92}H_{138}N_8Si_4$, 1468,0192; found, 1468,0148.

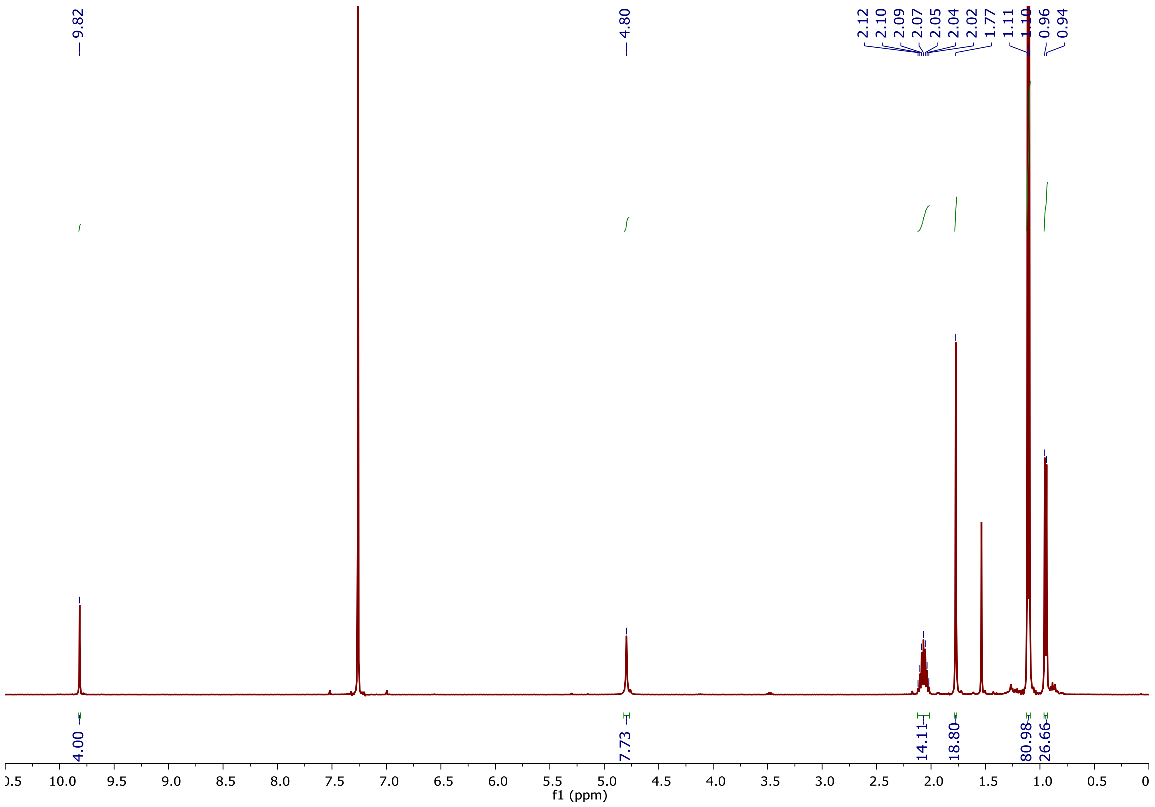

Figure S17. ^{1}H NMR (400 MHz, $CDCl_3$) of **NR-6**.

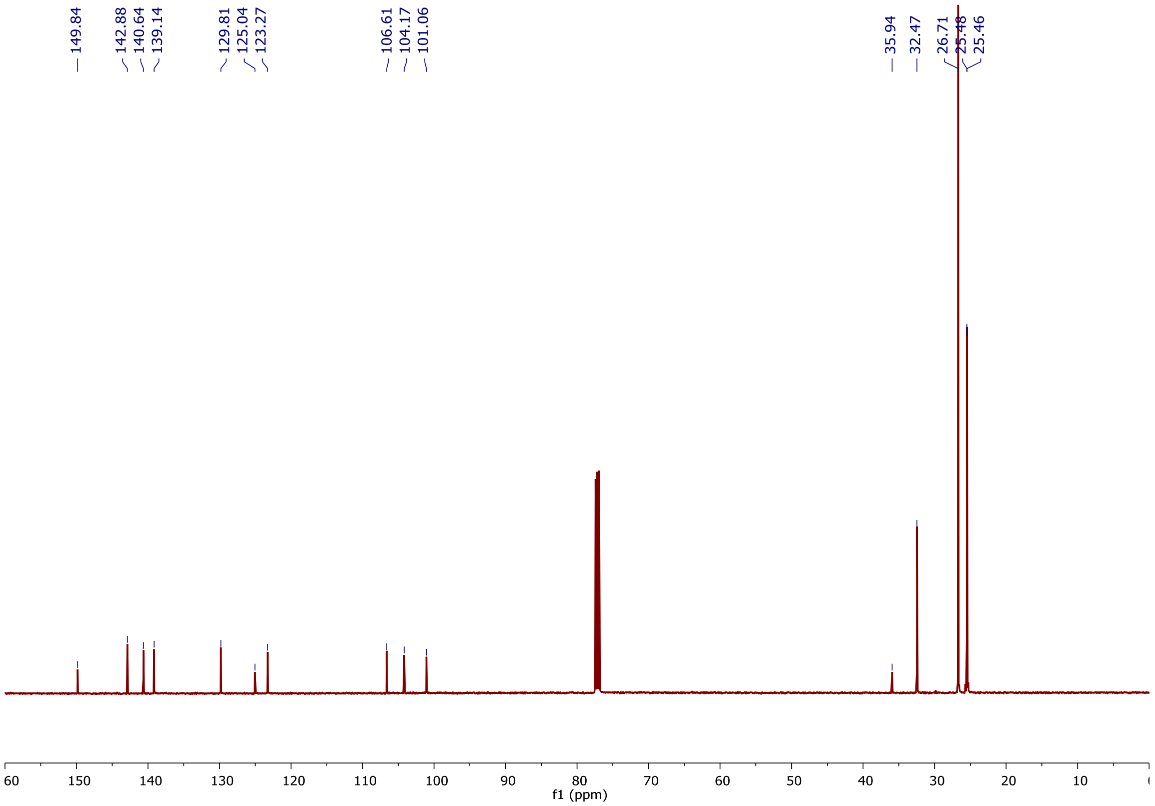

Figure S18. ^{13}C NMR (126 MHz, $CDCl_3$) of **NR-6.**

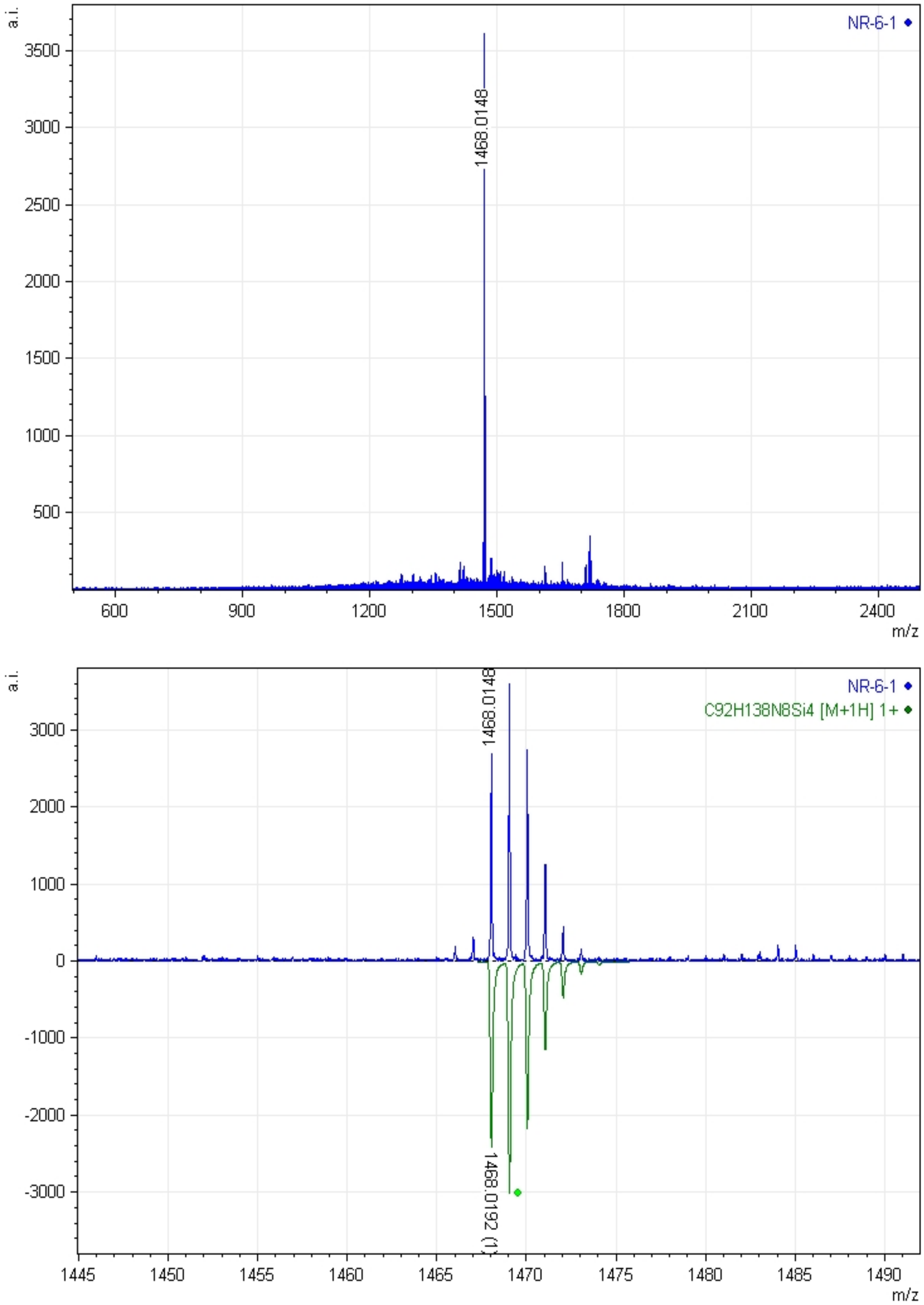

Figure S19. Full (top) and zoomed (bottom) MALDI-TOF mass spectrum of **NR-6**.

Synthesis of Compound NR-12:

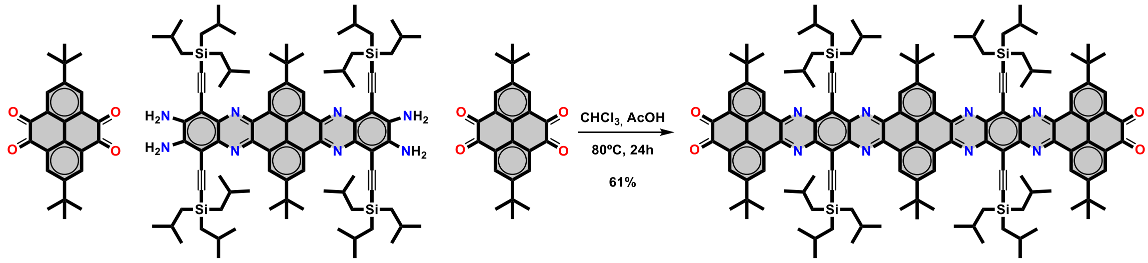

B NR-6 B NR-12

Molecular Weight: 2145,32

An oven dried sealable vial was charged with **NR-6** (20.00 mg, 0.013 mmol, 1 eq.) and pyrene-4,5,9,10-tetraketones (20.39 mg, 0.054 mmol, 4 eq.). Subsequently, anhydrous chloroform (3 mL) and glacial acetic acid (1 mL) were added and the vial was sealed with a PTFE/Silicone septum. The reaction was left to stir 24 hours at 80°C. After being cooled to room temperature, the crude product was extracted with chloroform (3 x 30 mL) and the organic layer was washed with water using a separatory funnel. The combined organic phase was concentrated and the crude product was purified by silica-gel column chromatography eluting with hexane – chloroform – ethyl acetate (3 : 2 : 0.05) to afford the product **C** (17.00 mg, 61%). From the column unreacted pyrene-4,5,9,10-tetraketones (5.00 mg) was collected.

^{1}H NMR (400 MHz, $CDCl_3$) δ 10.00 (s, 1H), 9.86 (d, *J* = 2.2 Hz, 1H), 8.71 (d, *J* = 2.2 Hz, 1H), 2.14 (m, *J* = 13.3, 6.4 Hz, 5H), 1.88 (s, 5H), 1.67 (s, 9H), 1.12 (d, *J* = 6.5 Hz, 30H).

^{13}C NMR (126 MHz, $CDCl_3$) δ 180.27, 153.05, 151.68, 145.20, 143.37, 142.57, 142.10, 131.23, 130.58, 130.38, 129.68, 129.61, 127.74, 122.32, 113.63, 102.33, 36.13, 35.84, 32.45, 31.67, 29.86, 26.66, 25.44, 25.30.

HRMS (MALDI-TOF) $[M+Ag]^+$ Calculated for $C_{140}H_{174}N_8O_4Si_4$, 2250,1777; found, 2250,1851.

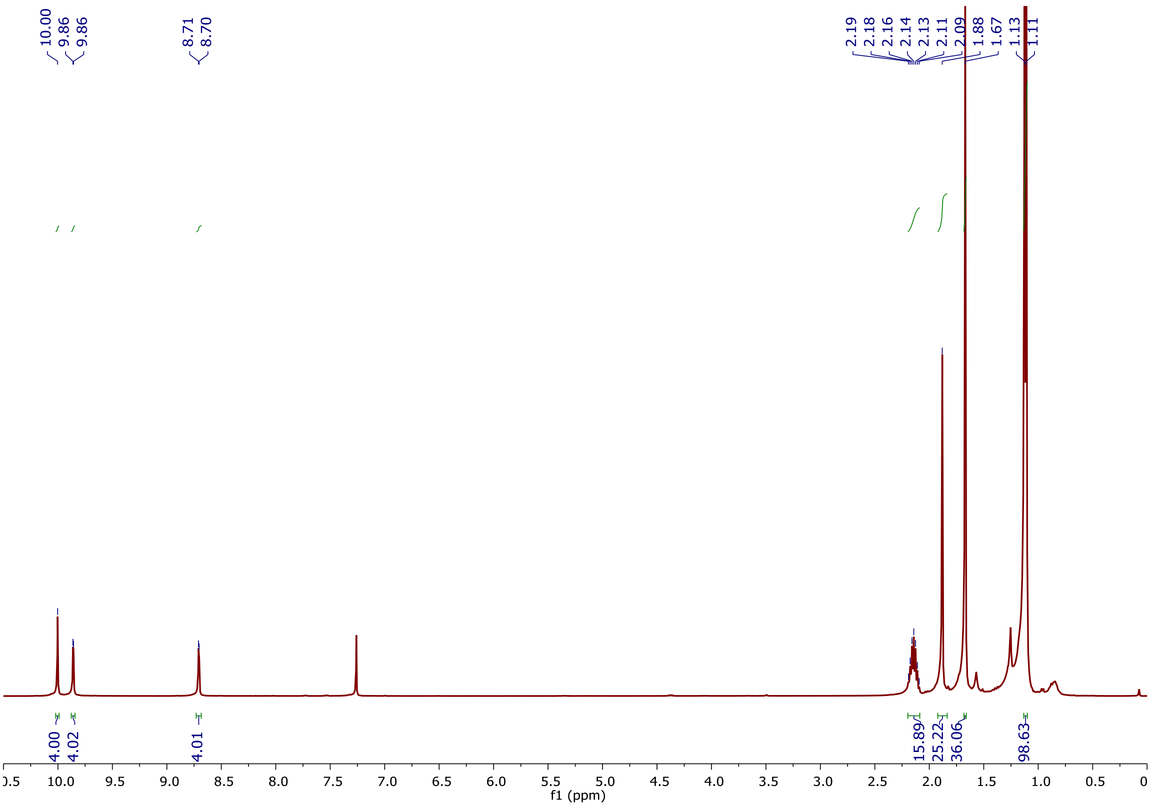

Figure S20. ^{1}H NMR (400 MHz, $CDCl_3$) of **NR-12**.

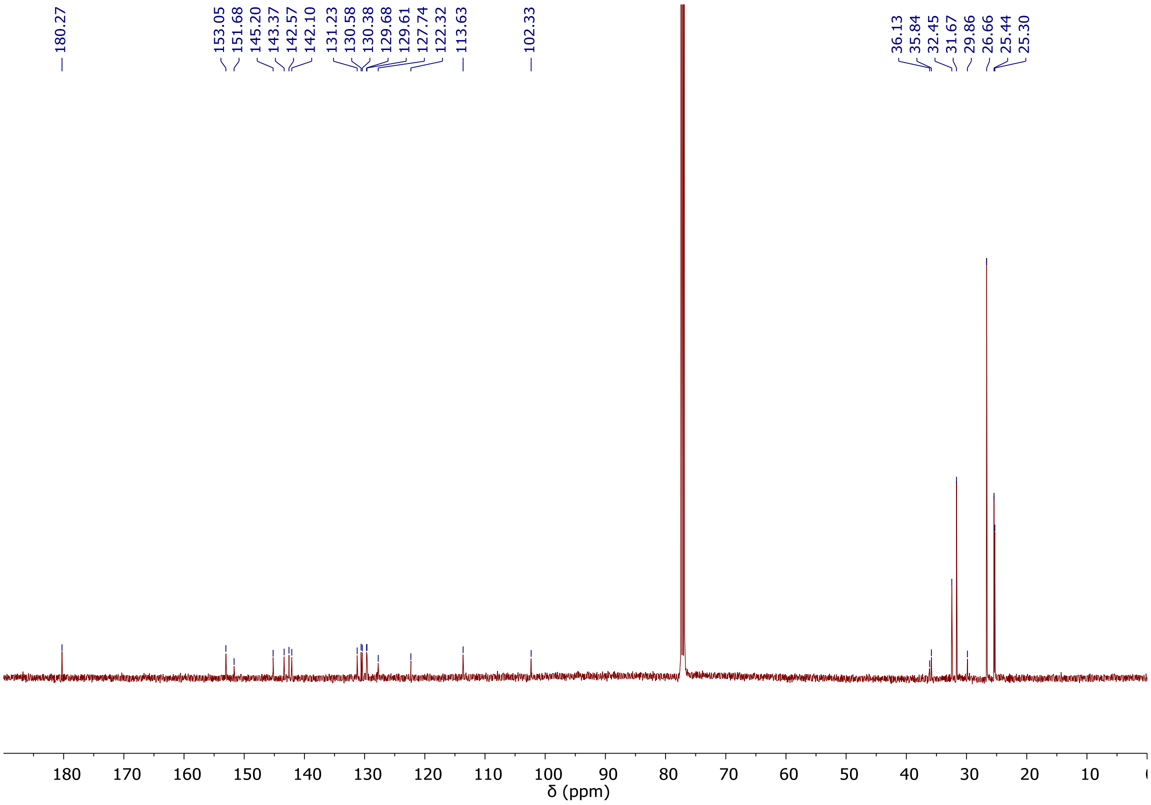

Figure S21. ^{13}C NMR (126 MHz, $CDCl_3$) of **NR-12**

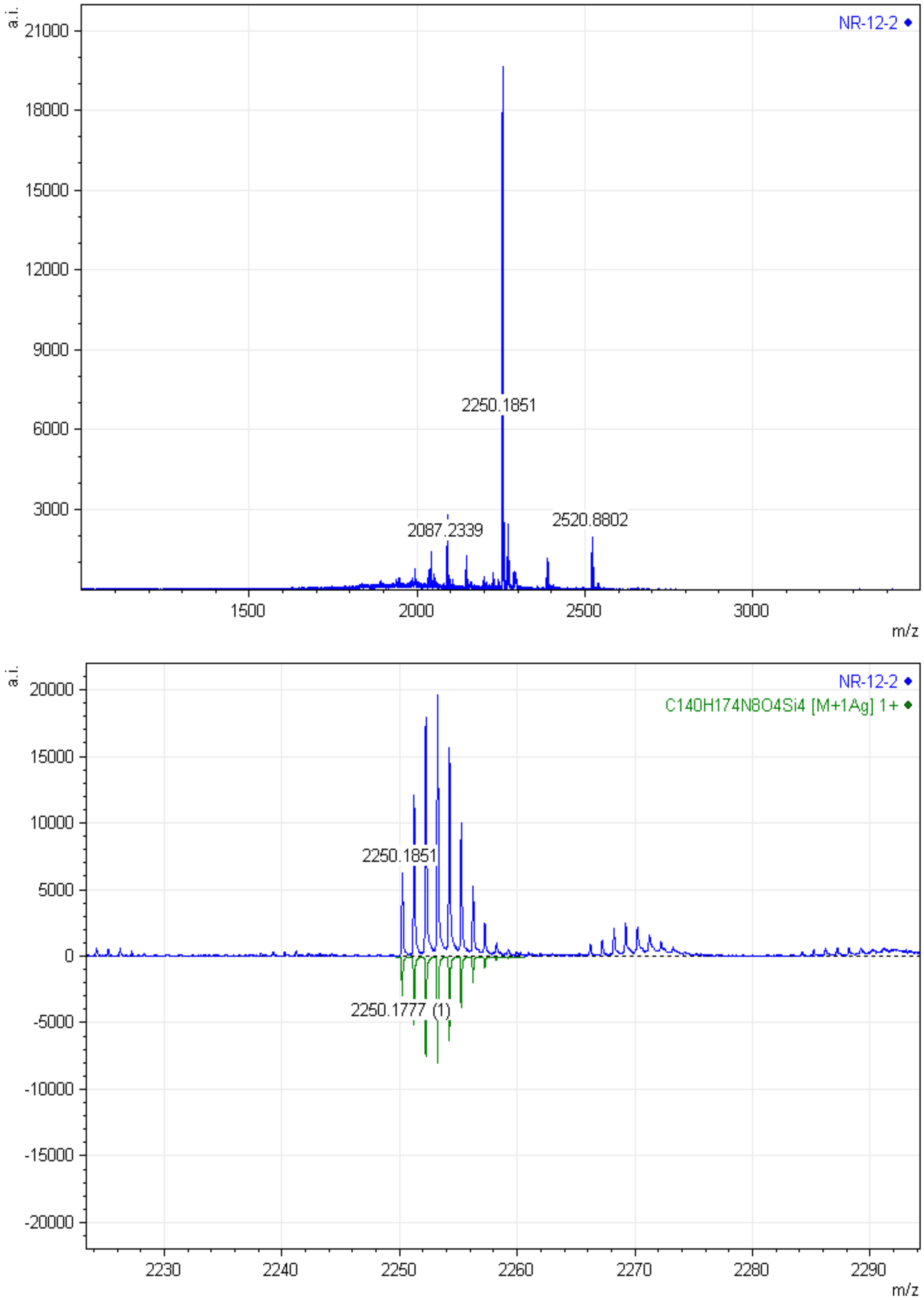

Figure S22. Full (top) and zoomed (bottom) MALDI-TOF mass spectrum of **NR-12**.

Synthesis of Compound NR-16:

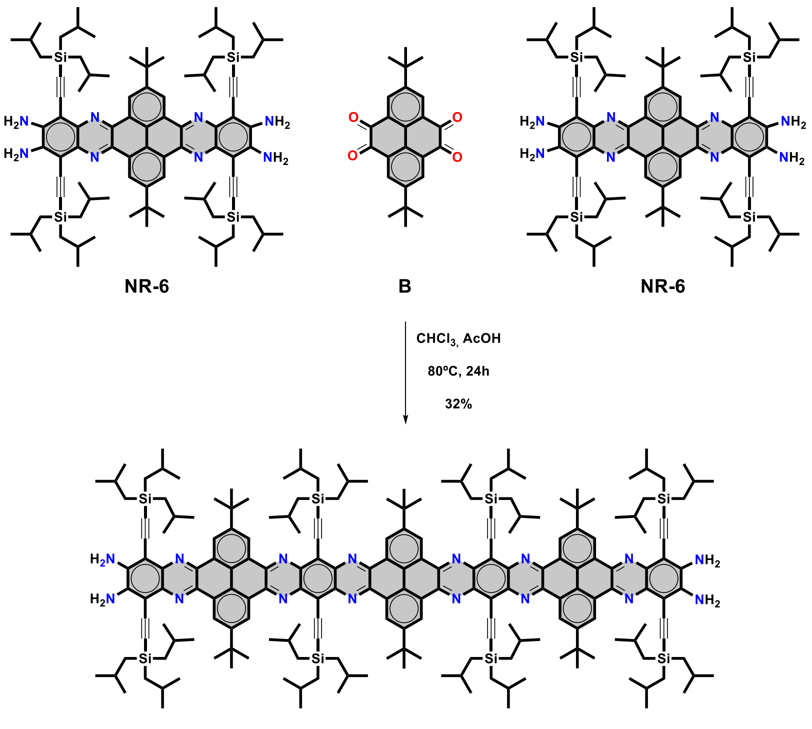

Pyrene-4,5,9,10-tetraketones (2.00 mg, 0.0053 mmol, 1.00 eq.) and **NR-6** (31.37 mg, 0.021 mmol, 4.00 eq.) were dissolved in anhydrous chloroform (3 mL) and glacial acetic acid (1 mL). The solution was taken in a sealed reaction vial and the reaction was stirred at 80°C for 24 h. After being cooled to room temperature, the crude product was extracted with chloroform (3 x 30 mL). The organic layer was concentrated under reduced pressure and the wanted product was purified by column chromatography (3:2 hexane - dichloromethane).
The product **NR-16** (5.50 mg, 32%) was obtained as a red solid.

^{1}H NMR (400 MHz, $CDCl_3$) δ 10.02 (s, 1H), 9.94 (d, *J* = 2.1 Hz, 1H), 9.92 (d, *J* = 2.2 Hz, 1H), 4.86 (s, 2H), 2.19 (m, *J* = 13.3, 6.7 Hz, 3H), 2.08 (m, *J* = 13.3, 6.7 Hz, 3H), 1.90 (s, 5H), 1.85 (s, 10H), 1.14 (m, *J* = 6.6, 5.4 Hz, 44H), 0.97 (d, *J* = 7.0 Hz, 6H).

^{13}C NMR (126 MHz, $CDCl_3$) δ 150.64, 145.57, 144.76, 143.19, 142.47, 142.31, 140.09, 139.32, 130.27, 129.80, 129.33, 127.32, 126.21, 125.60, 125.38, 121.91, 112.51, 106.84, 104.00, 102.68, 100.89, 36.04, 32.53, 32.49, 31.11, 29.85, 26.72, 25.48, 25.34.

HRMS (MALDI-TOF) Calculated for $C_{208}H_{290}N_{16}Si_8$, 3343,0376; found, 3343,0218.

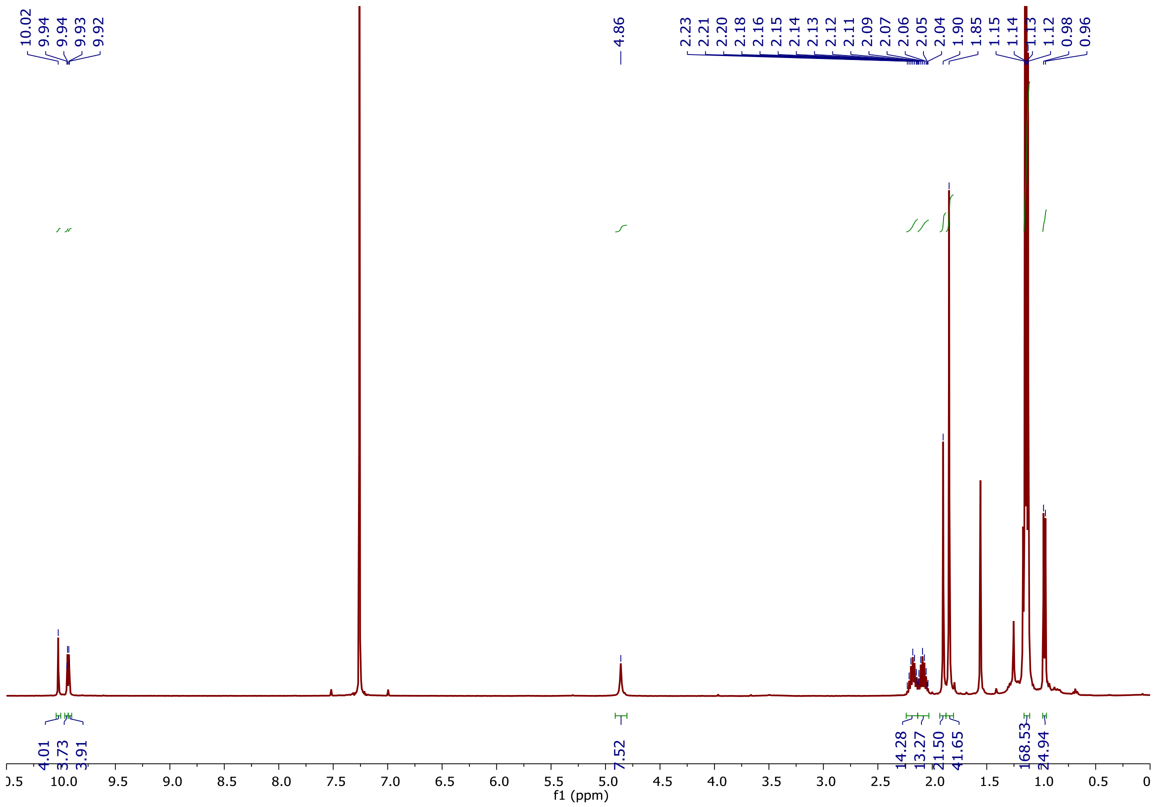

Figure S23. ^{1}H NMR (400 MHz, $CDCl_3$) of **NR-16**.

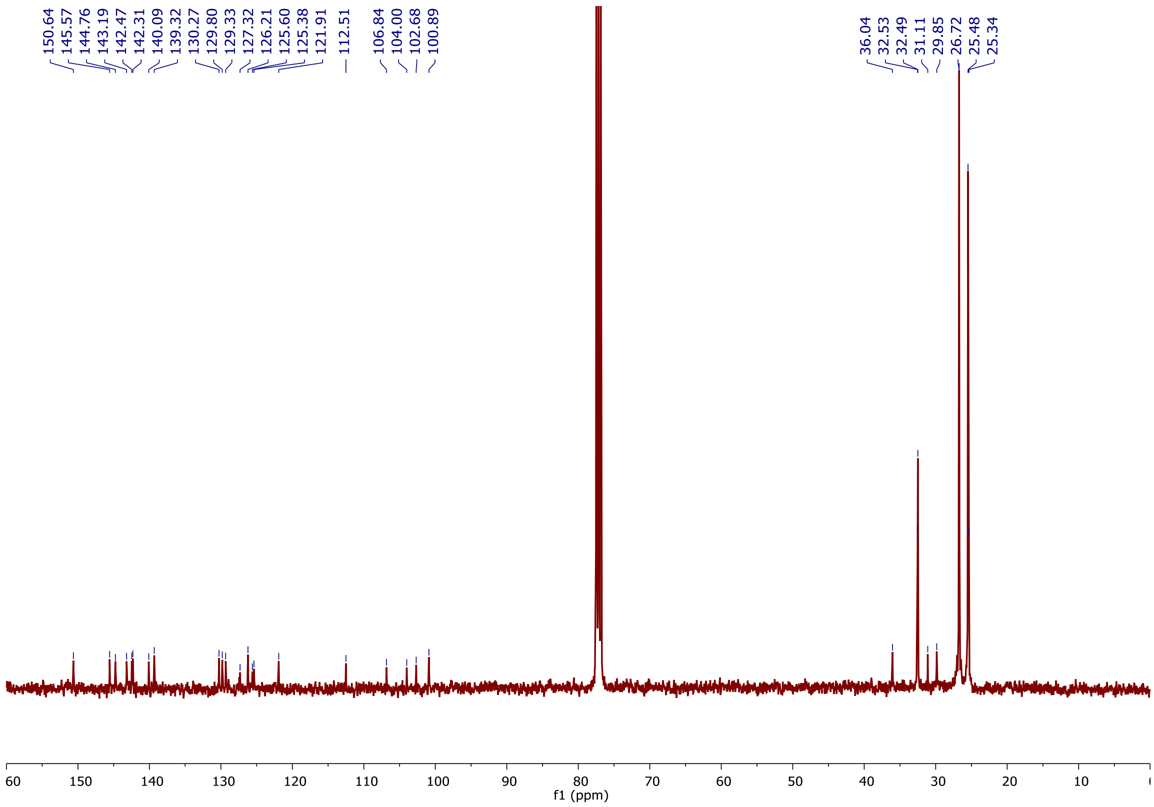

Figure S24. ^{13}C NMR (126 MHz, $CDCl_3$) of **NR-16**.

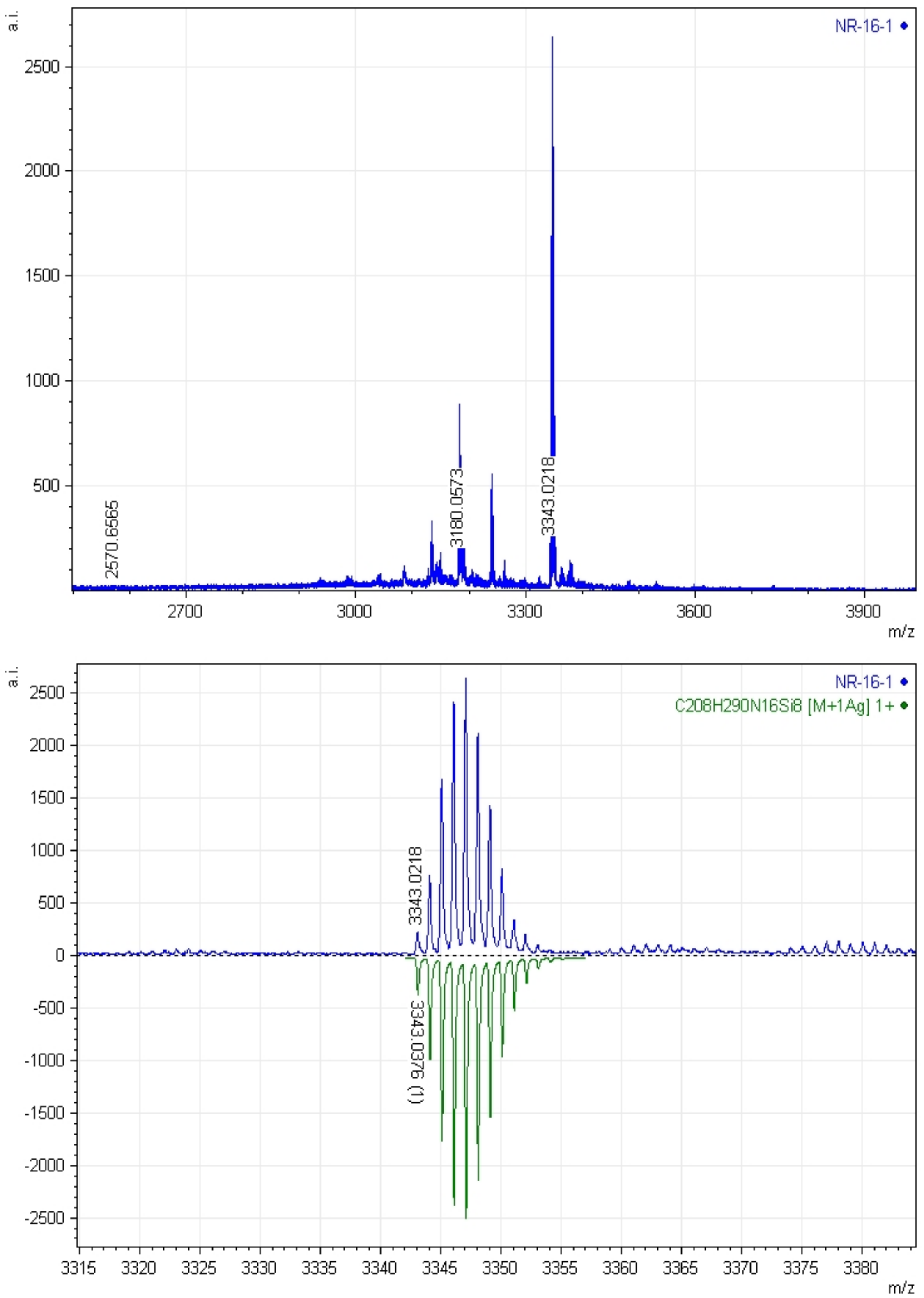

Figure S25. Full (top) and zoomed (bottom) MALDI-TOF mass spectrum of **NR-16**.

Synthesis of Compound NR-26:

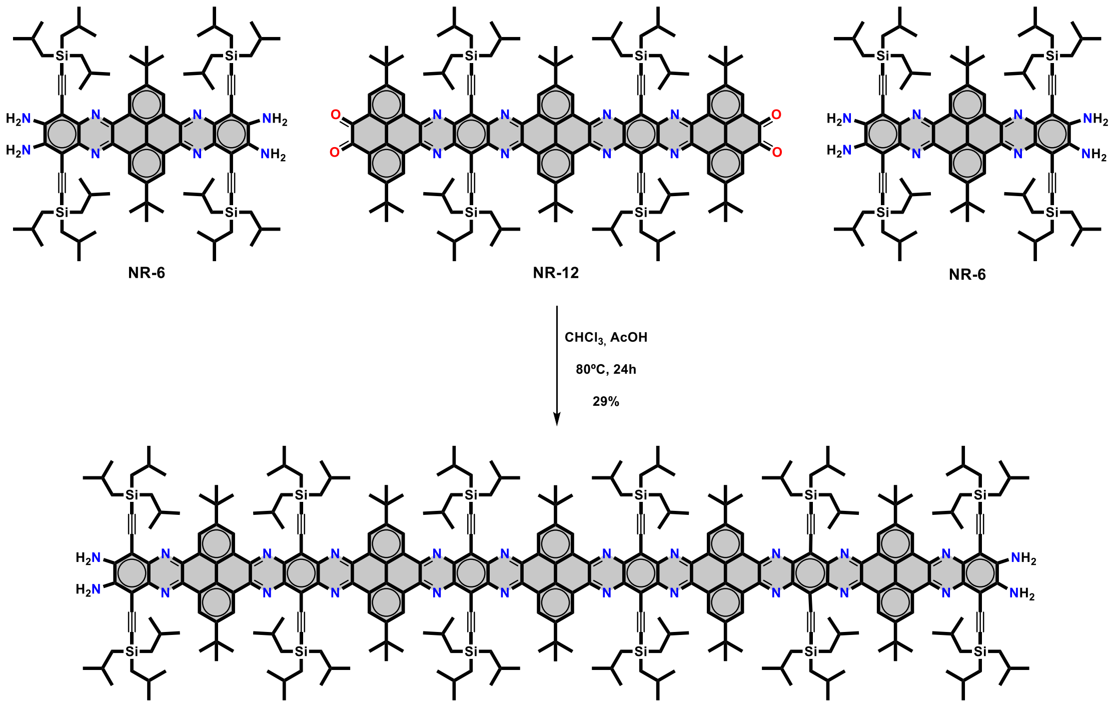

An oven dried sealable vial was charged with **NR-6** (43.80 mg, 0.0298 mmol, 4 eq.) and tetraketone based **NR-12** (16.00 mg, 0.0074 mmol, 1 eq.). Subsequently, anhydrous chloroform (3 mL) and glacial acetic acid (1 mL) were added and the vial was sealed with a PTFE/Silicone septum. The reaction was left to stir 24 hours at 80°C. After being cooled to room temperature, the crude product was extracted with chloroform (3 x 30 mL) and the organic layer was washed with water using a separatory funnel. The combined organic phase was concentrated and the crude product was purified by silica-gel column chromatography eluting with hexane – dichloromethane (6.5 : 3.5) to afford **NR-26** (11.00 mg, 29%). From the column unreacted compound **NR-6** (10.00 mg) was collected.

^{1}H NMR (400 MHz, $CDCl_3$) δ 10.05 (s, 1H), 10.04 (s, 1H), 9.95 (d, *J* = 2.1 Hz, 0H), 9.93 (d, *J* = 2.1 Hz, 0H), 4.87 (s, 1H), 2.20 (m, *J* = 13.4, 6.7 Hz, 2H), 2.08 (m, *J* = 13.2, 6.6 Hz, 1H), 1.92 (d, *J* = 3.0 Hz, 6H), 1.85 (s, 3H), 1.16 (m, *J* = 6.9, 6.5 Hz, 29H), 0.97 (d, *J* = 6.9 Hz, 2H).

^{13}C NMR (126 MHz, $CDCl_3$) δ 151.49, 150.67, 145.61, 144.94, 144.73, 143.21, 142.46, 142.33, 140.11, 139.35, 130.31, 129.82, 129.34, 127.51, 126.24, 125.65, 125.39, 122.11, 121.94, 112.90, 112.56, 106.85, 104.04, 102.69, 100.93, 36.15, 36.05, 32.54, 32.50, 32.08, 31.09, 29.86, 29.52, 26.73, 25.50, 25.39, 22.85, 14.28.

HRMS (MALDI-TOF) Calculated for $C_{324}H_{442}N_{24}Si_{12}$, 5114,1603; found, 5114,1584.

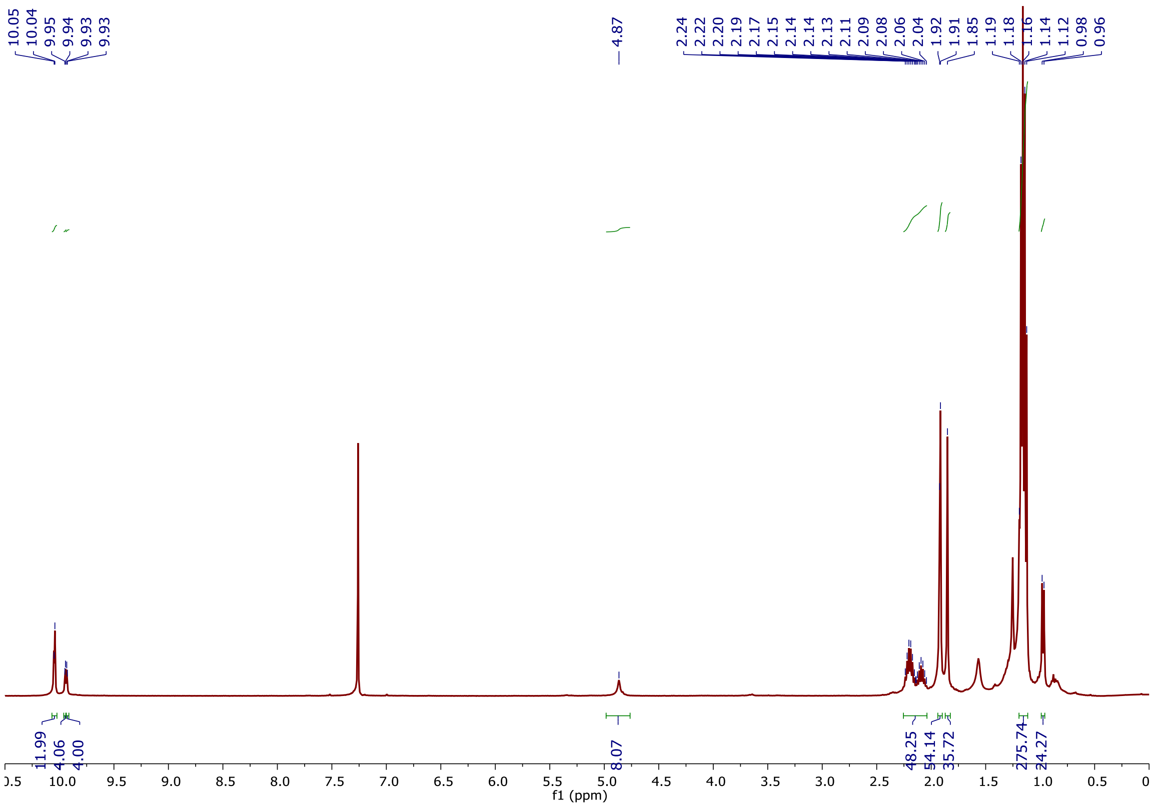

Figure S26. ^{1}H NMR (400 MHz, $CDCl_3$) of **NR-26**.

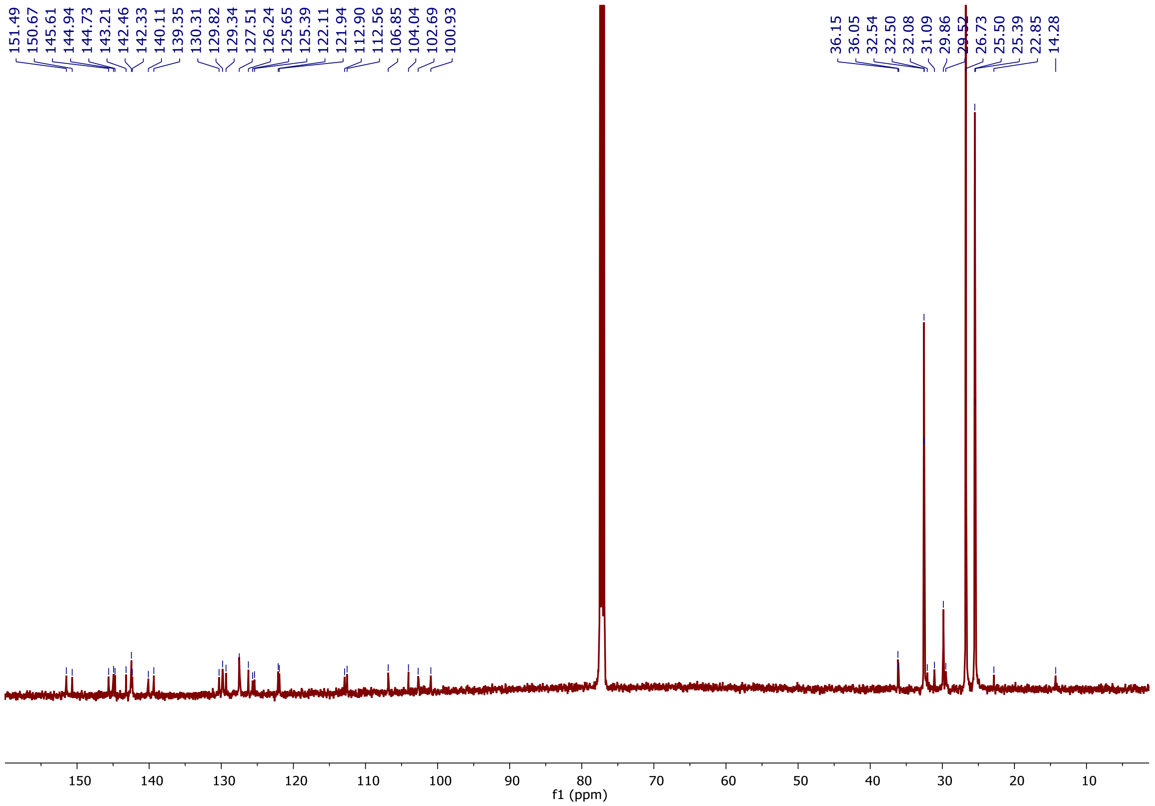

Figure S27. ^{13}C NMR (126 MHz, $CDCl_3$) of **NR-26**.

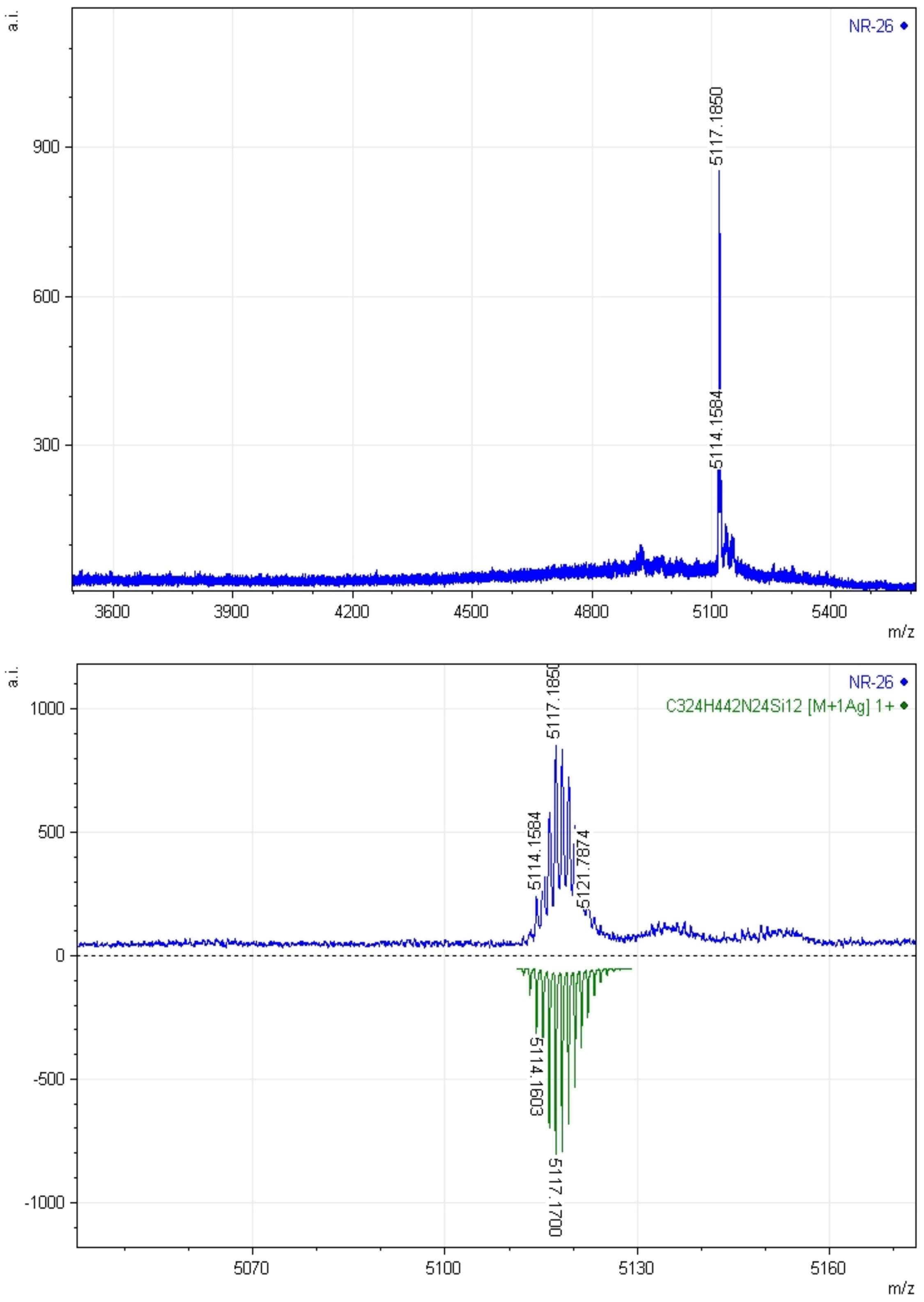

Figure S28. Full (top) and zoomed (bottom) MALDI-TOF mass spectrum of **NR-16**.

References

1. Dubey, R. K.; Melle-Franco, M.; Mateo-Alonso, A., Twisted Molecular Nanoribbons with up to 53 Linearly-Fused Rings. *J. Am. Chem. Soc.* **2021,** *143* (17), 6593-6600.
2. Marco, A. B.; Cortizo-Lacalle, D.; Perez-Miqueo, I.; Valenti, G.; Boni, A.; Plas, J.; Strutyński, K.; De Feyter, S.; Paolucci, F.; Montes, M.; Khlobystov, A. N.; Melle-Franco, M.; Mateo-Alonso, A., Twisted Aromatic Frameworks: Readily Exfoliable and Solution-Processable Two-Dimensional Conjugated Microporous Polymers. *Angew. Chem. Int. Ed.* **2017,** *56* (24), 6946-6951.
3. Hu, J.; Zhang, D.; Harris, F. W., Ruthenium(III) Chloride Catalyzed Oxidation of Pyrene and 2,7-Disubstitued Pyrenes: An Efficient, One-Step Synthesis of Pyrene-4,5-diones and Pyrene-4,5,9,10-tetraones. *J. Org. Chem.* **2004,** *70* (2), 707-708.
4. Xu, B.; Tao, N. J., Measurement of Single-Molecule Resistance by Repeated Formation of Molecular Junctions. *Science* **2003,** *301* (5637), 1221-1223.
5. Li; Hihath, J.; Chen, F.; Masuda, T.; Zang, L.; Tao, Thermally Activated Electron Transport in Single Redox Molecules. *J. Am. Chem. Soc.* **2007,** *129* (37), 11535-11542.
6. Hines, T.; Díez-Pérez, I.; Nakamura, H.; Shimazaki, T.; Asai, Y.; Tao, N., Controlling Formation of Single-Molecule Junctions by Electrochemical Reduction of Diazonium Terminal Groups. *J. Am. Chem. Soc.* **2013,** *135* (9), 3319-3322.
7. Aragonès, A. C.; Aravena, D.; Cerdá, J. I.; Acís-Castillo, Z.; Li, H.; Real, J. A.; Sanz, F.; Hihath, J.; Ruiz, E.; Díez-Pérez, I., Large Conductance Switching in a Single-Molecule Device through Room Temperature Spin-Dependent Transport. *Nano Lett.* **2016,** *16* (1), 218-226.
8. Balogh, Z.; Makk, P.; Halbritter, A., Alternative types of molecule-decorated atomic chains in Au–CO–Au single-molecule junctions. *Beilstein J. Nanotechnol.* **2015,** *6*, 1369-1376.
